# Treatable inherited metabolic disorders causing intellectual disability: 2021 review and digital app

**DOI:** 10.1186/s13023-021-01727-2

**Published:** 2021-04-12

**Authors:** Eva M. M. Hoytema van Konijnenburg, Saskia B. Wortmann, Marina J. Koelewijn, Laura A. Tseng, Roderick Houben, Sylvia Stöckler-Ipsiroglu, Carlos R. Ferreira, Clara D. M. van Karnebeek

**Affiliations:** 1 Department of Pediatrics, Amsterdam UMC, Amsterdam, The Netherlands; 2 Department of Pediatrics, Radboud Center for Mitochondrial Medicine, Radboud University Medical Center, Nijmegen, The Netherlands; 3 University Children’s Hospital, Paracelsus Medical University, Salzburg, Austria; 4 On Behalf of United for Metabolic Diseases, Amsterdam, The Netherlands; 5 Division of Biochemical Diseases, Department of Pediatrics, BC Children’s Hospital, Vancouver, BC V6H 3V4 Canada; 6 Health2Media, Amsterdam, The Netherlands; 7 National Human Genome Research Institute, National Institutes of Health, Bethesda, MD USA; 8 Department of Pediatrics - Metabolic Diseases, Amalia Children’s Hospital, Geert Grooteplein 10, Radboud University Medical Center, 6525 GA Nijmegen, The Netherlands

**Keywords:** Inborn error of metabolism, Metabolic disorders, Management, Therapy, Epilepsy, Intellectual developmental disorders, Diet, Pharmacological, Nutraceutical, Diagnostic, Outcomes, Evidence

## Abstract

**Background:**

The Treatable ID App was created in 2012 as digital tool to improve early recognition and intervention for treatable inherited metabolic disorders (IMDs) presenting with global developmental delay and intellectual disability (collectively ‘treatable IDs’). Our aim is to update the 2012 review on treatable IDs and App to capture the advances made in the identification of new IMDs along with increased pathophysiological insights catalyzing therapeutic development and implementation.

**Methods:**

Two independent reviewers queried PubMed, OMIM and Orphanet databases to reassess all previously included disorders and therapies and to identify all reports on Treatable IDs published between 2012 and 2021. These were included if listed in the International Classification of IMDs (ICIMD) and presenting with ID as a major feature, and if published evidence for a therapeutic intervention improving ID primary and/or secondary outcomes is available. Data on clinical symptoms, diagnostic testing, treatment strategies, effects on outcomes, and evidence levels were extracted and evaluated by the reviewers and external experts. The generated knowledge was translated into a diagnostic algorithm and updated version of the App with novel features.

**Results:**

Our review identified 116 treatable IDs (139 genes), of which 44 newly identified, belonging to 17 ICIMD categories. The most frequent therapeutic interventions were nutritional, pharmacological and vitamin and trace element supplementation. Evidence level varied from 1 to 3 (trials, cohort studies, case–control studies) for 19% and 4–5 (case-report, expert opinion) for 81% of treatments. Reported effects included improvement of clinical deterioration in 62%, neurological manifestations in 47% and development in 37%.

**Conclusion:**

The number of treatable IDs identified by our literature review increased by more than one-third in eight years. Although there has been much attention to gene-based and enzyme replacement therapy, the majority of effective treatments are nutritional, which are relatively affordable, widely available and (often) surprisingly effective. We present a diagnostic algorithm (adjustable to local resources and expertise) and the updated App to facilitate a swift and accurate workup, prioritizing treatable IDs. Our digital tool is freely available as Native and Web App (www.treatable-id.org) with several novel features. Our Treatable ID endeavor contributes to the Treatabolome and International Rare Diseases Research Consortium goals, enabling clinicians to deliver rapid evidence-based interventions to our rare disease patients.

**Supplementary Information:**

The online version contains supplementary material available at 10.1186/s13023-021-01727-2.

## Background

The past decade has seen revolutionary changes in the diagnosis and discovery of inherited metabolic disorders (IMDs), as well as development of new treatments [1]. Trials with small patient numbers remain challenging, but new methods for trial design, e.g., using natural history data as controls and crossover n-of-1 designs, have advanced our ability to determine whether treatments are effective or not [2]. What have these advances meant over the past decade for the treatment options of global developmental delay (DD according to the definition in Table 1A) and intellectual disability (ID), which is characterized by limitations starting before the age of 18 years in both intellectual functioning (IQ less than 70) and adaptive behavior. Our systematic literature review changed paradigms for this previously considered untreatable condition affecting 1–3% of the world’s population with substantial comorbidity, high lifetime costs, and emotional burden by identifying 81 treatable IDs in 2012, which increased to 89 in 2014 [3, 4]. With our review, we aimed to increase awareness and avoid the diagnostic and treatment delays so often suffered by rare diseases patients, with 25% of patients waiting 5 to 30 years for a diagnosis alone [5]. Now once again we address this medical gap and present an updated list of all Treatable IDs, which we define as IMDs which present with global developmental delay (DD) or ID yet are amenable to interventions targeting pathophysiology (e.g., nutraceutical, pharmacological, surgical, etc.) if initiated in a timely fashion.

**Table 1:** A and B Definitions and search terms

A. Definitions used in literature review
Global developmental delay (DD): applied to age < 5 years; significant delay (= performance two standard deviations or more below the mean on age-appropriate, standardised norm-referenced testing) in two or more developmental domains including gross/fine motor skills, speech/language, cognition, social/personal, activities of daily living [53] Intellectual disability (ID): applied to age ≥ 5 years and manifesting before age 18 years, historically referred to as ‘mental retardation’; intellectual functioning level (IQ) less than 70 to 75 and significant limitations in two or more adaptive skills [54, 55] Inherited Metabolic Disorder (IMD): impairment of specific enzymes or biochemical pathways that is intrinsic to the pathomechanism. The presence of an abnormal metabolite is no longer a prerequisite [6]. This term excludes endocrine disorders such as hypothyroidism Causal of ID/DD: sufficient evidence in literature from bench and/or clinical research to make a pathophysiological relationship between IMD and ID/DD highly likely Treatable ID: if a particular therapeutic modality is capable of preventing or improving ID/DD phenotype, or halting/slowing neurocognitive decline (with acceptable adverse effects) in the IMD, i.e., positively influencing the ‘outcome measures’ Treatment strategies: Nutritional therapy, vitamin & trace element, enzyme replacement therapy, hematopoietic stem cell transplant, solid organ transplantation, pharmacological therapy, gene-based therapy, other (e.g., hemodialysis) Outcome measure/effect: A = improves psychomotor/cognitive development/IQ, B = improves behavioural/psychiatric disturbance(s), C = prevents acute metabolic decompensation, D = prevents, halts, or slows clinical deterioration, E = improves neurological manifestations (incl. neuro-imaging), F = improves seizure/epilepsy control, G = improves systemic manifestations Levels of evidence: Level of evidence: Level 1a = systematic review of RCT's, 1b = individual RCT, 1c = ‘All or None’ [= (prolongation of) survival with therapy]; Level 2a = systematic review of cohort studies, 2b = individual cohort study, 2c = ‘Outcomes Research’ [focused on end results of therapy for chronic conditions, including functioning and quality of life]; Level 3 = systematic review of case– control studies; Level 4 = individual case–control study or case-series/report; Level 5 = expert opinion without critical appraisal; based on physiology, bench research or first principles. If only one patient was reported, we assigned level ‘4–5′ as a way to nuance the treatment effects
B. Terms used for search strategy in PubMed [56]
Developmental delay/intellectual disability: mental retardation, learning disorder(s), developmental disability/ disabilities, learning disability/disabilities, intellectual disability/disabilities, developmental delay, intelligence/classification, mentally disabled (persons), childhood/juvenile Alzheimer's, childhood/juvenile dementia, neurodegenerative disease Inherited Metabolic Disorder: metabolic disease(s), inborn error(s) of metabolism, metabolic disorder(s), metabolic condition(s), inherited metabolic disease(s), inherited metabolic disorder(s), biochemical disease(s) Treatment: treatment, management, therapy, cure, trial, (dietary) supplement, (dietary) restriction, diet, substrate inhibition, small molecule substrate reduction, enzyme replacement, vitamin(s), co-factor(s), bone marrow transplant, hematopoietic stem cell transplant, umbilical cord blood transplant(− ation), gene therapy

There are several developments that should be considered to place the current overview of Treatable IDs in perspective. First, the new all-inclusive definition of an IMD, proposed in 2015 by Morava et al*.* [6] and recently endorsed by the international metabolic community in the International Classification of Inherited Metabolic Disorders (ICIMD): ‘Any condition in which the impairment of a biochemical pathway is intrinsic to the pathophysiology of the disease, regardless of whether there are abnormalities in currently available biochemical laboratory tests’(http://www.icimd.org) [7]. The number of IMDs now exceeds 1400 [8]. Second, the practice change our diagnostic algorithm has inspired, with international professional societies now prioritizing IMDs in the diagnostic evaluation of patients with ID in whom the cause is not evident after a thorough clinical exam [9–11] The digital tool ‘Treatable ID’ has certainly given this innovation a boost [12] as its digital accessibility proved useful both for educational as well as practical purposes (e.g., Continuum Child Neurology [13]), especially in remote areas where metabolic expertise might not be available onsite. The Treatable ID App was created in 2012, and is freely accessible as a Web App via http://www.treatable-id.org and since 2016 as a Native App via the App Store/Google Play. The App is designed for a target audience of various specialists evaluating children presenting with ID, both clinicians and laboratory scientists from student to expert level [12]. The Treatable ID App has a steady audience. Over the past 8 years there have been over 75,000 different users for the web App and over 10,000 downloads of the native App. The Treatable ID App is also part of the Treatable Intellectual Disability Endeavor (TIDE) diagnostic protocol [14]. In the second tier of the TIDE algorithm, the Treatable ID App is incorporated to optimize selection of targeted metabolic workup [14]. Also, numerous clinical and commercial labs have requested access to the Treatable ID gene lists for their quick turnaround phenotype-driven (virtual) gene panels. Third, as shown in a retrospective study by Sayson et al*.*, using the Treatable ID algorithm can reduce costs and diagnostic delay for treatable IMDs underlying ID [15]. The same was shown in a prospective way, in our TIDE study which implemented the Treatable ID algorithm in 498 unexplained ID patients referred to a tertiary care centre (biochemical and clinical genetics as well as neurology departments) as add-on to clinical practice parameters at the time (2015). Indeed, this manuscript currently under review illustrates the presence of IMDs (6%) in this group of patients, even those without a classical multi-organ or degenerative phenotype (van Karnebeek et al*.* submitted) [16]. The fourth development is the remarkably large number of clinical trials with great promise, even pioneering gene-based therapy targeting the central nervous system, few of which however have made it to the real-world of reimbursed clinical care, and as such are not included here. Finally, despite the advances, the inequality in access to exome sequencing (ES) and other -omics technologies remains. Indeed, access determines the diagnostic approach; while for some countries and regions, metabolic testing is still the first tier, for others the exome-first approach has become standard of care [17]. Thus, algorithms must be tailored to local possibilities and expertise. Speed and accuracy are warranted as ‘time is brain’, i.e., early identification and intervention before irreversible damage is done [18]. Increasingly, therapy is center stage; even in the interpretation of genomic variants, response to therapy has been endorsed as a valuable criterion to determine pathogenicity [19]. The ultimate goal of creating a Treatabolome database comprising rare disease treatments at gene and variant levels was recently outlined [20]. Here, we contribute to this goal by presenting an updated state-of the-art overview of all treatable IDs along with a new version of the digital App freely accessible to professionals as well as patients, and suggest an updated diagnostic algorithm.

## Material and methods

Our main goals were: (1) to identify all IMDs presenting with DD and/or ID (collectively termed ID in this paper) as a major feature, which are amenable to treatment targeting pathophysiology, supported by evidence in the literature reported up to January 2021, and (2) to translate this information into an updated version of the Treatable ID digital App, as well as (3) a diagnostic algorithm to facilitate early detection of treatable IMD in patients presenting with unexplained ID.

### Literature review

For our literature review, we used a critical rather than a formal systematic literature review approach to answer the above stated question. We followed the Treatabolome approach as much as possible [20]. The systematic literature review in 2012 and its update in 2014 were used as a basis. Three independent reviewers (EH, CvK, SW) searched and critically appraised the literature, characterized the clinical and diagnostic recognition patterns as well as treatment modalities pertinent to the identified IMDs, and assessed the level of available evidence and effect of the various treatments on clinical outcome measures. The reviewers engaged in regular consensus meetings, and final decisions on any disagreements were reached by a majority vote of the reviewers plus an external expert (CRF).

### Identification of treatable IMDs causing ID

#### Literature search

Definitions of terms relevant for the search strategy and keywords for terms DD, ID, IMD, and treatment are shown in Table 1A and B. We searched PubMed, restricted to English language and publication in peer-reviewed journals (http://www.ncbi.nlm.nih.gov/pubmed; 1960–January 2021) in a two-step approach. First, the 89 IMDs included in the 2012 and 2014 database were reassessed under consideration of the additional literature published; second, new treatable IDs were identified and reviewed. Additionally, the reference lists of identified articles, Orphanet [21] and Online Mendelian Inheritance in Man (OMIM) [22] were queried with the same search terms, and experts in the field were approached to identify new treatable IDs. To ensure comprehensiveness of treatment modalities, we identified all relevant references reporting outcome/effect for each of the selected treatments and IMDs. We searched bibliographies of included articles as well as PubMed (1960–January 2021) combining as keywords all known names for each IMD (including gene and enzyme) with the relevant therapeutic modalities (Table 1B).

#### Outcomes and levels of evidence

The ideal outcome of therapy for a treatable ID is the improvement of IQ and related developmental scores. As improvement of co-morbid features such as epilepsy, neurologic, behavioral or psychiatric problems is often a prerequisite for improved cognitive outcomes, these were included as ‘secondary outcomes’. Levels of evidence were defined and applied according to Table 1A.

#### Effect(s) of treatments on outcome measures

Effect(s) of treatment outcomes were defined as shown in Table 1A. We included treatments if they had a direct effect on ID (improvement or stabilization), or if there was a reasonable expectation that ID would be improved by significant improvement of other symptoms—such as seizures or severe movement disorders—thus making development possible. Supportive treatments and treatments with improvement only of systemic symptoms that were not reasonably related to ID were not included. If a treatment was not effective for all reported patients, we defined that at least a third of the patients needed to show improvement to ensure inclusion of potential beneficial treatments. All decisions on inclusion and exclusion of IMDs and treatments were mutually agreed upon during a final consensus meeting. In the previous review, a classification about standard of care versus individual basis was assigned to each treatment; given the growing attention to personalized medicine in IMD, we do not feel this distinction appropriate for our current review.

#### Inclusion/exclusion criteria

We included IMDs where ID was a major clinical feature (present in more than 50% of reported patients) and for which evidence supported amenability to the defined treatments with a positive effect on outcomes in at least one-third of patients.

#### Data extraction

For all IMDs meeting the criteria of a Treatable ID, the following information was captured:

Name of disease; gene and inheritance pattern; name of biochemical deficiency; group of disorder; screening and specific tests; neurological and non-neurological symptoms (only characteristic, specific and consistent symptoms were noted, see http://www.treatable-ID.org); treatment strategies (see Table 1A for categorization); specific therapies; level of evidence for each treatment and treatment effect (see Table 1A). Names and groups of disorders were based on the most recent ICIMD [7]. Disorders caused by pathogenic variants in multiple genes were reported as separate entries if there were meaningful differences in phenotype or treatment. For practical and user-friendliness purposes, if there were no meaningful differences, genes were grouped or ‘lumped’ and considered as one IMD.

### Compliance to the FAIR guidelines for scientific data management and stewardship

We adhered as much as possible to the FAIR-compliant (findable, accessible, interoperable, reusable) template that will ultimately enable the building of a ‘Treatabolome’ database [20, 23]. We complied with the recommendations for a broad and inclusive literature search and the main elements of data extraction and data synthesis. The main deviation from the FAIR-compliant template in our review is that, due to the multitude of included IMDs, we did not perform a formal systematic review for each IMD. Furthermore, there are a few specific deviations from the template: we used OMIM gene/locus instead of phenotype OMIM numbers as we considered this approach more precise for the current review, and we did not specify all (contra)indications and marketing authorizations for each treatment due to of the multitude of included IMDs; this was outside the scope of this review.

### Diagnostic tests and algorithm

We used our literature review results to update our diagnosed algorithm proposed in 2014. During the past years, ES has become accepted as first line tier testing in many countries around the world. However, metabolic screening is still applied given the specificity and sensitivity of tests, the short turnaround times and relative affordability and availability [24]. Metabolic profiles can also serve as functional readout, and “deep metabolic phenotyping” can help in the interpretation of genetic data. Therefore, we included both strategies in our algorithm to facilitate a practical guide for biochemical and genetic/genomic diagnosis. We first assessed which tests are necessary to identify each of the conditions. Accordingly, we grouped the IMDs into those diagnosed via ‘metabolic screening tests’ (1st Tier) versus IMDs diagnosed via ‘single test per single disease’ (2nd Tier) approach. First tier screening tests were defined as tests in blood and urine which are readily available in biochemical laboratories in most developed countries. Metabolic tests in the 2nd tier group evaluate Treatable IDs for which biochemical markers are difficult to interpret, and/or conventional diagnostic approach requires an invasive procedure or poorly accessible test (i.e., only performed in few centres worldwide). Furthermore, we analyzed which IMDs have no (reliable) biomarker profile and require primary molecular or (targeted) ES analysis. This approach with different strategies and tiers was then translated into a step-wise algorithm.

### Treatable ID App design and development

The Treatable ID App was created in 2012 and later updated based on the 2012 and 2014 literature reviews [3, 4]. In 2021, the Treatable ID App has been updated and improved extensively both in content and design. The content is updated based on our 2021 literature search with updated and increased links to useful resources for each IMD. The design has been adapted to 2021 standards with a quick and solid interface. Both the Web App and the Native App use the same database which is an improvement over the previous version. As a result, new information can be added easily and this will ensure the content is always up to date. The database is built in Oracle.

The Web App can be used in all major browsers and the Native app can be downloaded from the App Store and Google Play. The creation of the Treatable ID App is supported and funded by the ‘Metakids Foundation’ in The Netherlands [25].

## Results

### Literature review

#### Treatable IDs

Our first systematic review identified 81 treatable IDs [3] and was updated with another 8 disorders in 2014 [4]. From these 89 disorders, our current literature search led to exclusion of 20 treatable IDs, because of insufficient evidence for effect of treatment on established outcome measures (n = 10), ID no longer considered a major clinical feature (n = 8), metabolic defect not causative of ID (n = 1) and duplicate disorder (n = 1). The disorders are listed in Additional File 1: Table 1.

Of the 69 remaining treatable IDs we grouped the following IMDs according to the method described: (1) Glycine encephalopathy due to aminomethyltransferase (*AMT*) or glycine decarboxylase *(GLDC*) deficiency; (2) Electron transfer flavoprotein subunit alpha (*ETFA*)/subunit beta (*ETFB*)/dehydrogenase deficiency (*ETFDH*); 3) Branched-chain ketoacid dehydrogenase E1 alpha (*BCKDHA*)/beta (*BCKDHB*)/E2 (*DBT*) deficiency; (4) Propionic acidemia due to propionyl-CoA carboxylase subunit alpha (*PCCA*)/beta (*PCCB*) deficiency; (5) Niemann-Pick disease type C1 (*NPC1*)/C2 (*NPC2*); (6) Pyruvate dehydrogenase E1 alpha (*PDHA1*)/beta (*PDHB*)/E2 (*DLAT*)/E3 (*DLD*)/E3BP (*PDHX*) deficiency; (7) ATP-sensitive potassium channel regulatory/pore-forming subunit superactivity (*ABCC8/KCNJ11*); (8) Mitochondrial myopathy, encephalopathy, lactic acidosis and stroke-like episodes (*MTTL1/MTTQ/MTTH/MTTK/MTTC/MTTS1/MTND1*/*MTND5*/*MTND6*/*MTTS2*).

We further ‘split’ two previously grouped (‘lumped’) disorders into five separate disease entities: 1) Coenzyme Q5 methyltransferase deficiency, 2) Coenzyme Q8A (*ADCK*3) deficiency, 3) Methylmalonic aciduria and homocystinuria, cblD type, 4) Homocystinuria, cblDv1 type, 5) Methylmalonic aciduria, cblDv2 type. This led to a final list of 72 treatable IDs that were already included in the 2014 database. Our literature search for “new” treatable IDs identified 44 disorders, combining to a total number of 116 treatable IDs included in this 2021 update and shown in Table 2 (new disorders are marked with an *).

**Table 2:** Overview of 116 treatableIDs and diagnostic tests

Group of disorder	Name of disorder	Gene(s)	Orphanet#	OMIM gene/locus#	HPO#	Diagnostic test
Congenital disorders of glycosylation	SLC35A2-CDG*	*SLC35A2 (X-linked)*	356,961	314,375	7355	Serum transferrin/N-glycan profiling
Congenital disorders of glycosylation	SLC35C1-CDG*	*SLC35C1-CDG (AR)*	99,843	605,881	55,343	Serum transferrin/N-glycan profiling
Congenital disorders of glycosylation	PMM2-CDG*	*PMM2 (AR)*	79,318	601,785	5373	Serum transferrin/N-glycan profiling
Congenital disorders of glycosylation	PIGA-CDG*	*PIGA (X-linked)*	–	311,770	–	Molecular testing
Congenital disorders of glycosylation	PIGM-CDG*	*PIGM (AR)*	83,639	610,273	93,183	Molecular testing
Congenital disorders of glycosylation	PIGO-CDG*	*PIGO (AR)*	247,262	614,730	84,720	Molecular testing
Disorders of amino acid metabolism	Arginase deficiency (synonym: argininemia)	*ARG1 (AR)*	90	608,313	383	Plasma amino acids Plasma ammonia
Disorders of amino acid metabolism	Argininosuccinate lyase deficiency	*ASL (AR)*	23	608,310	435	Plasma amino acids Plasma ammonia
Disorders of amino acid metabolism	Argininosuccinate synthetase deficiency (synonym: citrullinemia type 1)	*ASS1 (AR)*	247,525	603,470	445	Plasma amino acids Plasma ammonia
Disorders of amino acid metabolism	Citrin deficiency	*SLC25A13 (AR)*	247,582	603,859	10,165	Plasma amino acids
Disorders of amino acid metabolism	Methionine synthase deficiency (synonym: homocystinuria-megaloblastic anemia, cblG type)	*MTR (AR)*	2170	156,570	4548	Plasma total homocysteine
Disorders of amino acid metabolism	Carbamoyl phosphate synthetase 1 deficiency	*CPS1 (AR)*	147	608,307	1373	Plasma amino acids
Disorders of amino acid metabolism	Mitochondrial sulfur dioxygenase deficiency (synonym: ethylmalonic encephalopathy)	*ETHE1 (AR)*	51,188	608,451	23,474	Urine organic acids Plasma acylcarnitines Molecular testing
Disorders of amino acid metabolism	Glutaryl-CoA dehydrogenase deficiency (synonym: glutaric aciduria type 1)	*GCDH (AR)*	25	608,801	2639	Plasma acylcarnitines Urine organic acids
Disorders of amino acid metabolism	Mitochondrial ornithine transporter deficiency (synonym: hyperornithinemia-hyperammonemia-homocitrullinuria syndrome)	*SLC25A15 (AR)*	415	603,861	10,116	Plasma amino acids Plasma ammonia
Disorders of amino acid metabolism	Cystathionine beta-synthase deficiency (synonym: classic homocystinuria)	*CBS (AR)*	394	613,381	875	Urine organic acids Plasma total homocysteine
Disorders of amino acid metabolism	Isovaleryl-CoA dehydrogenase deficiency (synonym: isovaleric acidemia)	*IVD (AR)*	33	607,036	3712	Plasma acylcarnitines Urine organic acids
Disorders of amino acid metabolism	N-acetylglutamate synthase deficiency	*NAGS (AR)*	927	608,300	162,417	Plasma amino acids Plasma ammonia
Disorders of amino acid metabolism	Glycine encephalopathy due to aminomethyltransferase (AMT) or glycine decarboxylase (GLDC) deficiency (synonym: nonketotic hyperglycinemia)	*AMT*/*GLDC (AR)*	407	238,310 (AMT), 238,300 (GLDC)	275 (AMT), 2731 (GLDC)	Plasma amino acids CSF amino acids
Disorders of amino acid metabolism	Branched-chain ketoacid dehydrogenase E1 alpha (BCKDHA)/beta (BCKDHB)/E2 (DBT) deficiency (synonym: maple syrup urine disease type 1a (BCKDHA)/2 (DBT); Dihydrolipoyl transacylase deficiency (DBT))	*BCKDHA*/*BCKDHB/ DBT (AR)*	268,145, 268,162, 268,184	608,348 (BCKDHA), 248,611 (BCKDHB), 248,610 (DBT)	593 (BCKDHA), 594 (BCKDHB), 1629 (DBT)	Plasma amino acids
Disorders of amino acid metabolism	Methylmalonic aciduria due to methylmalonyl-CoA mutase deficiency	*MMUT (AR)*	289,916, 79,312	609,058	4594	Plasma acylcarnitines Urine organic acids Blood lactate Plasma ammonia
Disorders of amino acid metabolism	Ornithine transcarbamylase deficiency	*OTC (X-linked)*	664	300,461	5009	Plasma amino acids Urine organic acids
Disorders of amino acid metabolism	Phenylalanine hydroxylase deficiency (synonym: phenylketonuria)	*PAH (AR)*	79,254	612,349	5053	Plasma amino acids
Disorders of amino acid metabolism	3-phosphoglycerate dehydrogenase deficiency	*PHGDH (AR)*	79,351	606,879	26,227	Plasma amino acids CSF amino acids
Disorders of amino acid metabolism	Phosphoserine aminotransferase deficiency	*PSAT1 (AR)*	284,417	610,936	29,968	Plasma amino acids CSF amino acids
Disorders of amino acid metabolism	Propionic acidemia due to propionyl-CoA carboxylase subunit alpha (PCCA)/beta (PCCB) deficiency	*PCCA*/*PCCB (AR)*	35	232,000 (PCCA), 232,050 (PCCB)	5095 (PCAA), 5096 (PCCB)	Plasma acylcarnitines Urine organic acids Blood lactate Plasma ammonia
Disorders of amino acid metabolism	Phosphoserine phosphatase deficiency	*PSPH (AR)*	79,350	172,480	5723	Plasma amino acids CSF amino acids
Disorders of amino acid metabolism	Tyrosine aminotransferase deficiency (synonyms: tyrosinemia type 2; Richner-Hanhart syndrome)	*TAT (AR)*	28,378	613,018	6898	Plasma amino acids Urine organic acids
Disorders of amino acid metabolism	Branched-chain ketoacid dehydrogenase kinase deficiency*	*BCKDK (AR)*	308,410	614,901	10,295	Plasma amino acids
Disorders of amino acid metabolism	Carbonic anhydrase VA deficiency	*CA5A (AR)*	401,948	114,761	763	Plasma amino acids Plasma ammonia blood lactate molecular testing
Disorders of amino acid metabolism	3-hydroxyisobutyryl-CoA hydrolase deficiency (synonym: beta-hydroxyisobutyryl-CoA deacylase deficiency)*	*HIBCH (AR)*	88,639	250,620	26,275	Urine organic acids Molecular testing
Disorders of amino acid metabolism	Mitochondrial short-chain enoyl-CoA hydratase 1 deficiency*	*ECHS1 (AR)*	255,241	616,277	1892	Urine organic acids Molecular testing
Disorders of amino acid metabolism	S-adenosylhomocysteine hydrolase deficiency*	*AHCY (AR)*	88,618	613,752	191	Plasma amino acids Plasma total homocysteine
Disorders of amino acid metabolism	Glutamine synthetase deficiency*	*GLUL (AR)*	71,278	610,015	2752	Plasma amino acids CSF amino acids
Disorders of carbohydrate metabolism	GLUT1 deficiency	*SLC2A1 (AD)*	71,277	138,140	6513	CSF other (CSF:blood glucose ratio)
Disorders of complex molecule degradation	Alpha-mannosidase deficiency	*MAN2B1 (AR)*	309,282, 309,288	609,458	4125	Urine oligosaccharides**
Disorders of complex molecule degradation	Aspartylglucosaminidase deficiency	*AGA (AR)*	93	613,228	175	Urine oligosaccharides**
Disorders of complex molecule degradation	Iduronate sulfatase deficiency (synonym: Hunter syndrome)	*IDS (X-linked)*	217,085	300,823	3423	Urine glycosaminoglycans**
Disorders of complex molecule degradation	Alpha-iduronidase deficiency (synonyms: Hurler syndrome [severe]; Scheie syndrome [attenuated])	*IDUA (AR)*	93,473	252,800	3425	Urine glycosaminoglycans**
Disorders of complex molecule degradation	Arylsulfatase A deficiency	*ARSA (AR)*	309,256, 309,263, 309,271	607,574	410	Enzymatic testing (Arylsulfatase A)
Disorders of complex molecule degradation	Niemann-Pick disease type C1 (NPC1)/C2 (NPC2)	*NPC1*/*NPC2 (AR)*	646	607,623 (NPC1), 601,015 (NPC2)	4864 (NPC1), 10,577 (NPC2)	Plasma (oxy-)sterols
Disorders of complex molecule degradation	Beta-glucuronidase deficiency (synonym: Sly syndrome)	*GUSB (AR)*	584	611,499	2990	Urine glycosaminoglycans**
Disorders of complex molecule degradation	Tripeptidyl-peptidase 1 deficiency (synonym: CLN2 disease)*	*TPP1 (AR)*	228,349	607,998	1200	Enzymatic testing (Tripeptidyl-peptidase 1)
Disorders of complex molecule degradation	Alpha-fucosidase deficiency*	*FUCA1 (AR)*	349	230,000	2517	Urine glycosaminoglycans**
Disorders of complex molecule degradation	CLN7 disease*	*MFSD8 (AR)*	228,366	611,124	256,471	Molecular testing
Disorders of energy substrate metabolism	Arginine:glycine amidinotransferase (AGAT) deficiency	*GATM (AR)*	35,704	602,360	2628	(Plasma/) urine creatine & guanidinoacetate
Disorders of energy substrate metabolism	Creatine transporter deficiency	*SLC6A8 (X-linked)*	52,503	300,036	6535	Urine creatine & guanidinoacetate
Disorders of energy substrate metabolism	Guanidinoacetate methyltransferase deficiency	*GAMT (AR)*	382	601,240	2593	Urine creatine & guanidinoacetate
Disorders of energy substrate metabolism	Pyruvate dehydrogenase E1 alpha (PDHA1)/beta (PDHB)/E2 (DLAT)/E3 (DLD)/E3BP (PDHX) deficiency	*PDHA1 (X-linked)*/*PDHB*/*DLAT*/*DLD*/*PDHX (AR)*	79,243 (PDHA1), 255,138 (PDHB), 79,244 (DLAT), 2394 (DLD), 255,182 (PDHX)	300,502 (PDHA1), 179,060 (PDHB), 608,770 (DLAT), 238,331 (DLD), 608,769 (PDHX)	5160 (PDHA1), 5162 (PDHB), 1737 (DLAT), 1738 (DLD), 8050 (PDHX)	CSF other (lactate:pyruvate ratio) other (blood lactate:pyruvate ratio)
Disorders of energy substrate metabolism	Pyruvate dehydrogenase phosphatase deficiency*	*PDP1 (AR)*	79,246	605,993	54,704	Plasma amino acids Urine organic acids
Disorders of fatty acid, carnitine, and ketone body metabolism	Mitochondrial acetoacetyl-CoA thiolase deficiency	*ACAT1 (AR)*	134	607,809	38	Urine organic acids
Disorders of fatty acid, carnitine, and ketone body metabolism	Electron transfer flavoprotein subunit alpha (ETFA)/subunit beta (ETFB)/dehydrogenase deficiency (ETFDH) (synonym: glutaric acidemia type 2A/2B/2C; multiple acyl-CoA dehydrogenase deficiency type 2A/2B/2C)	*ETFA*/*ETFB*/*ETFDH (AR)*	26,791	608,053 (ETFA), 130,410 (ETFB), 231,675 (ETFDH)	2108 (ETFA), 2109 (ETFB), 2110 (ETFDH)	Plasma acylcarnitines Urine organic acids
Disorders of fatty acid, carnitine, and ketone body metabolism	3-hydroxy-3-methylglutaryl-CoA lyase deficiency	*HMGCL (AR)*	20	613,898	3115	Urine organic acids
Disorders of fatty acid, carnitine, and ketone body metabolism	Mitochondrial 3-hydroxy-3-methylglutaryl-CoA synthase deficiency	*HMGCS2 (AR)*	35,701	600,234	3158	Urine organic acids
Disorders of fatty acid, carnitine, and ketone body metabolism	Epsilon-N-trimethyllysine hydroxylase deficiency*	*TMLHE (X-linked)*		300,777	55,217	Plasma acylcarnitines
Disorders of lipid metabolism	X-linked adrenoleukodystrophy	*ABCD1 (X-linked)*	139,396, 139,396	300,371	215	Plasma very long chain fatty acids
Disorders of lipid metabolism	Sterol 27-hydroxylase deficiency (synonym: cerebrotendinous xanthomatosis)	*CYP27A1 (AR)*	909	606,530	1593	Other (plasma cholestanol)
Disorders of lipid metabolism	7-dehydrocholesterol reductase deficiency	*DHCR7 (AR)*	818	602,858	1717	Plasma (oxy-)sterols
Disorders of mitochondrial cofactor biosynthesis	Coenzyme Q5 methyltransferase deficiency	*COQ5 (AR)*		616,359	84,274	Molecular testing***
Disorders of mitochondrial cofactor biosynthesis	Coenzyme Q8A (ADCK3) deficiency	*COQ8A (AR)*	139,485	606,980	56,997	Molecular testing
Disorders of nucleobase, nucleotide and nucleic acid metabolism	Isoleucyl-tRNA synthetase 1 deficiency*	*IARS1 (AR)*	541,423	600,709	3376	Molecular testing
Disorders of nucleobase, nucleotide and nucleic acid metabolism	Leucyl-tRNA synthetase 1 deficiency*	*LARS1 (AR)*	370,088	151,350	51,520	Molecular testing
Disorders of nucleobase, nucleotide and nucleic acid metabolism	Phenylalanyl-tRNA synthetase subunit beta deficiency*	*FARSB (AR)*	178,506	609,690	10,056	Molecular testing
Disorders of nucleobase, nucleotide and nucleic acid metabolism	Seryl-tRNA synthetase 1 deficiency*	*SARS1 (AR)*	88,616	607,529	6301	Molecular testing
Disorders of nucleobase, nucleotide and nucleic acid metabolism	Methionyl-tRNA synthetase 1 deficiency*	*MARS1 (AR)*	397,735, 401,835, 440,427	156,560	4141	Molecular testing
Disorders of nucleobase, nucleotide and nucleic acid metabolism	Phosphoribosylpyrophosphate synthetase deficiency*	*PRPS1 (X-linked)*	1187	311,850	5631	Urine purines & pyrimidines
Disorders of nucleobase, nucleotide and nucleic acid metabolism	CAD trifunctional protein deficiency*	*CAD (AR)*	448,010	114,010	790	Molecular testing
Disorders of peptide and amine metabolism	NRF2 superactivity (synonym: immunodeficiency, developmental delay, and hypohomocysteinemia)*	*NFE2L2 (AD)*		600,492	4780	Plasma total homocysteine
Disorders of trace elements and metals	Hereditary ceruloplasmin deficiency	*CP (AR)*	48,818	117,700	1356	Copper & ceruloplasmin
Disorders of trace elements and metals	Copper-transporting ATPase subunit alpha deficiency (synonyms: Menkes disease [severe]; occipital horn syndrome [milder])	*ATP7A (AR)*	565	300,011	538	copper & Ceruloplasmin
Disorders of trace elements and metals	Copper-transporting ATPase subunit beta deficiency (synonym: Wilson disease)	*ATP7B (AR)*	905	606,882	540	Copper & ceruloplasmin
Disorders of trace elements and metals	SLC39A8 deficiency*	*SLC39A8 (AR)*	468,699	608,732	64,116	Other (serum zinc, sialotransferrins, manganese, CSF lactate)
Disorders of trace elements and metals	MEDNIK syndrome	*AP1S1 (AR)*	171,851	609,313	1174	Copper & ceruloplasmin Molecular testing
Disorders of vitamin and cofactor metabolism	Thiamine transporter 2 deficiency (synonym: biotin-thiamine-responsive basal ganglia disease)	*SLC19A3 (AR)*	65,284, 199,348	606,152	80,704	Molecular testing
Disorders of vitamin and cofactor metabolism	Biotinidase deficiency	*BTD (AR)*	79,241	609,019	686	Enzymatic testing (Biotinidase)
Disorders of vitamin and cofactor metabolism	Folate receptor alpha deficiency (synonym: neurodegeneration due to cerebral folate transport deficiency)	*FOLR1 (AR)*	217,382	136,430	2348	CSF other (methyltetrahydrofolate)
Disorders of vitamin and cofactor metabolism	Methylmalonic aciduria, cblA type	*MMAA (AR)*	79,310	607,481	166,785	Urine organic acids
Disorders of vitamin and cofactor metabolism	Methylmalonic aciduria, cblB type	*MMAB (AR)*	79,311	607,568	326,625	Urine organic acids
Disorders of vitamin and cofactor metabolism	Methylmalonic aciduria and homocystinuria, cblC type	*MMACHC (AR)*	79,282	609,831	25,974	Urine organic acids Plasma total homocysteine
Disorders of vitamin and cofactor metabolism	Methylmalonic aciduria and homocystinuria, cblD type	*MMADHC (AR)*	79,283	611,935	27,249	urine organic acids Plasma total homocysteine
Disorders of vitamin and cofactor metabolism	Homocystinuria, cblDv1 type	*MMADHC (AR)*	308,380	611,935	27,249	Plasma total homocysteine
Disorders of vitamin and cofactor metabolism	Methylmalonic aciduria, cblDv2 type	*MMADHC (AR)*	308,442	611,935	27,249	Urine organic acids
Disorders of vitamin and cofactor metabolism	Methionine synthase reductase deficiency (synonym: homocystinuria-megaloblastic anemia, cblE type)	*MTRR (AR)*	2169	603,568	4552	Plasma total homocysteine
Disorders of vitamin and cofactor metabolism	Methylmalonic aciduria and homocystinuria, cblF type	*LMBRD1 (AR)*	79,284	612,625	55,788	Urine organic acids Plasma total homocysteine
Disorders of vitamin and cofactor metabolism	Methylmalonic aciduria and homocystinuria, cblJ type*	*ABCD4 (AR)*	369,955	603,214	5826	Urine organic acids Plasma total homocysteine
Disorders of vitamin and cofactor metabolism	Dihydropteridine reductase deficiency	*QDPR (AR)*	226	612,676	5860	CSF neurotransmitters Other (biopterin loading test)
Disorders of vitamin and cofactor metabolism	Autosomal recessive GTP cyclohydrolase 1 deficiency	*GCH1 (AR)*	2102	600,225	2643	CSF neurotransmitters
Disorders of vitamin and cofactor metabolism	Holocarboxylase synthetase deficiency	*HLCS (AR)*	79,242	609,018	3141	Urine organic acids
Disorders of vitamin and cofactor metabolism	Cyclic pyranopterin monophosphate synthase deficiency (synonym: molybdenum cofactor deficiency type A)	*MOCS1 (AR)*	308,386	603,707	4337	Urine purines & pyrimidines Urine sulfites/S-sulfocysteine, (serum uric acid)
Disorders of vitamin and cofactor metabolism	5,10-methylenetetrahydrofolate reductase deficiency	*MTHFR (AR)*	395	607,093	4524	Plasma amino acids Plasma total homocysteine
Disorders of vitamin and cofactor metabolism	6-pyruvoyl-tetrahydropterin synthase deficiency	*PTS (AR)*	13	612,719	5805	CSF neurotransmitters Other (biopterin loading test)
Disorders of vitamin and cofactor metabolism	Alpha-aminoadipic semialdehyde dehydrogenase deficiency (synonym: pyridoxine-dependent epilepsy)	*ALDH7A1 (AR)*	3006	107,323	501	Other (plasma P6C, urine alpha-aminoadipic semialdehyde)
Disorders of vitamin and cofactor metabolism	Sepiapterin reductase deficiency	*SPR (AR)*	70,594	182,125	6697	CSF neurotransmitters Other (biopterin loading test)
Disorders of vitamin and cofactor metabolism	5,10-methenyltetrahydrofolate synthetase deficiency (synonym: 5-formyltetrahydrofolate cycloligase deficiency)*	*MTHFS (AR)*	-	604,197	10,588	CSF neurotransmitters CSF other (tetrahydrofolate & neopterin)
Disorders of vitamin and cofactor metabolism	Dihydrofolate reductase deficiency	*DHFR (AR)*	319,651	126,060	1719	CSF neurotransmitters CSF other (tetrahydrofolate)
Disorders of vitamin and cofactor metabolism	Sodium-dependent multivitamin transporter deficiency*	*SLC5A6 (AR)*	–	604,024	8884	molecular testing
Disorders of vitamin and cofactor metabolism	Pyridoxamine 5′-phosphate oxidase deficiency*	*PNPO (AR)*	79,096	603,287	55,163	CSF amino acids CSF other (vitamins) Molecular testing
Disorders of vitamin and cofactor metabolism	Thiamine pyrophosphokinase deficiency*	*TPK1 (AR)*	293,955	614,458	27,010	CSF neurotransmitters Urine organic acids Blood lactate
Disorders of vitamin and cofactor metabolism	NAD(P)HX epimerase deficiency*	*NAXE (AR)*	555,407	608,862	128,240	Molecular testing Blood lactate
Disorders of vitamin and cofactor metabolism	Mitochondrial thiamine pyrophosphate transporter deficiency*	*SLC25A19 (AR)*	217,396	606,521	60,386	Molecular testing
Disorders of vitamin and cofactor metabolism	Transcobalamin II deficiency*	*TCN2 (AR)*	859	275,350	6948	Urine organic acids Plasma total homocysteine
Disorders of vitamin and cofactor metabolism	Proton-coupled folate transporter deficiency (synonym: hereditary folate malabsorption)*	*SLC46A1 (AR)*	90,045	229,050	113,235	Serum/RBC folate CSF other (folate) CSF neurotransmitters
Endocrine metabolic disorders	ATP-sensitive potassium channel regulatory/pore-forming subunit superactivity*	*ABCC8 (AD*/*AR)*/*KCNJ11 (AD)*	79,134	600,509	6833	Molecular testing
mtDNA-related disorders	Mitochondrial myopathy, encephalopathy, lactic acidosis and stroke-like episodes (MTTL1/MTTQ/MTTH/MTTK/MTTC/MTTS1/MTND1/MTND5/MTND6/MTTS2)	*MT-ND1*/*MT-ND4*/*MT-ND5*/*MT-ND6*/*MT-CO1*/*MT-CPO2*/*MT-CO3/ MT-TQ*/*MT-TH*/*MT-TL1*/*MT-TF*/*MT-TS1*/*MT-TS2*/*MT-TW (Mt)*	550	516,000 (MT-ND1), 516,003 (MT-ND4), 516,005 (MT-ND5), 516,006 (MT-ND6), 516,030 (MT-CO1), 516,040 (MT-CO2), 516,050 (MT-CO3), 590,030 (MT-TQ), 590,040 (MT-TH), 590,050 (MT-TL1), 590,070 (MT-TF), 590,080 (MT-TS1), 590,085 (MT-TS2), 590,095 (MT-TW)	4535 (MT-ND1), 4536 (MT-ND4), 4540 (MT-ND5), 4541 (MT-ND6), 4512 (MT-CO1), 4513 (MT-CO2), 4514 (MT-CO3), 4572 (MT-TQ), 4574 (MT-TS1), 4578 (MT-TW)	Molecular testing blood lactate
Neurotransmitter disorders	Succinic semialdehyde dehydrogenase deficiency	*ALDH5A1 (AR)*	22	610,045	7915	Urine organic acids
Neurotransmitter disorders	Tyrosine hydroxylase deficiency	*TH (AR)*	101,150	191,290	7054	CSF neurotransmitters
Neurotransmitter disorders	Vesicular monoamine transporter 2 deficiency	*SLC18A2 (AR)*	352,649	193,001	6571	CSF neurotransmitters
Neurotransmitter disorders	Aromatic L-amino acid decarboxylase deficiency*	*DDC (AR)*	35,708	107,930	1644	CSF neurotransmitters
Neurotransmitter disorders	Ionotropic glutamate receptor NMDA type subunit 1 dysregulation*	*GRIN1 (AD)*	-	138,249	2902	Molecular testing
Neurotransmitter disorders	Ionotropic glutamate receptor NMDA type subunit 2A dysregulation*	*GRIN2A (AD)*	289,266	138,253	2903	Molecular testing
Neurotransmitter disorders	Ionotropic glutamate receptor NMDA type subunit 2B dysregulation*	*GRIN2B (AD)*	-	138,252	2904	Molecular testing
Neurotransmitter disorders	Ionotropic glutamate receptor NMDA type subunit 2D superactivity*	*GRIN2D (AD)*	442,835	602,717	2906	Molecular testing
Neurotransmitter disorders	DNAJC12 deficiency*	*DNAJC12 (AR)*	508,523	617,384	56,521	Plasma amino acids CSF neurotransmitters
Nuclear-encoded disorders of oxidative phosphorylation	ACAD9 deficiency*	*ACAD9 (AR)*	99,901	611,126	28,976	other (muscle OXPHOS)
Other disorders of mitochondrial function	Mitochondrial aspartate-glutamate carrier isoform 1 deficiency (synonym: aralar deficiency)*	*SLC25A12 (AR)*	353,217	612,949	8604	Molecular testing
Other disorders of mitochondrial function	Mitochondrial aspartate aminotransferase deficiency*	*GOT2 (AR)*	-	138,150	2806	Plasma amino acids Plasma ammonia Blood lactate

Names and groups of disorders were based on the most recent International Conference on Inherited Metabolic Disorders (ICIMD) classification. OMIM = Online Mendelian Inheritance in Man®. HPO = Human Phenotype Ontology. Mode of inheritance for each gene is denoted as AD = autosomal dominant; AR = autosomal recessive; X-linked (dominant); Mt = mitochondrial. CSF = cerebrospinal fluid. * = new treatable ID (not included in previous database/review). ** = also identified by targeted enzyme testing. *** = consider white blood cells or muscle tissue CoQ10 analysis

**Table 3:** Overview of all causal therapies

Name of disorder	Treatment strategy	Treatment	Level of evidence	Effect of treatment	ReferenceS
3-hydroxy-3-methylglutaryl-CoA lyase deficiency	Nutritional therapy	Protein defined diet, avoid fasting, sick day management	5	C	[57]
3-hydroxyisobutyryl-CoA hydrolase deficiency (synonym: beta-hydroxyisobutyryl-CoA deacylase deficiency)	Nutritional therapy	Valine restriction	4	A, E, G	[58]
3-phosphoglycerate dehydrogenase deficiency	Nutritional therapy	L-Serine, glycine	4	D, E, F	[59]
5,10-methenyltetrahydrofolate synthetase deficiency (synonym: 5-formyltetrahydrofolate cycloligase deficiency)	Vitamin & trace element	5-methyltetrahydrofolate, methylcobalamin	5	E	[60]
5,10-methylenetetrahydrofolate reductase deficiency	Nutritional therapy	Carnitine, methionine	4	C, D, G	[61]
	Vitamin & trace element	Betaine, folate ±	4	C, D, G	[61]
6-pyruvoyl-tetrahydropterin synthase deficiency	Nutritional therapy	Phenylalanine-reduced diet ±	4	D, E	[62]
	Vitamin & trace element	Folinic acid ±	4	A, E, F	[62]
	Pharmacological therapy	L-dopa + carbidopa, 5-Hydroxytryptophan, sapropterin dihydrochloride (synthetic BH4) co-factor therapy	4	A, B, E, F, G	[62]
7-dehydrocholesterol reductase deficiency	Nutritional therapy	Cholesterol	4	G	[63, 64]
	Pharmacological therapy	Simvastatin	1b	B	[63, 64]
ACAD9 deficiency	Vitamin & trace element	Riboflavin	4	A, E	[65]
Alpha-aminoadipic semialdehyde dehydrogenase deficiency (synonym: pyridoxine-dependent epilepsy)	Nutritional therapy	Lysine restriction, arginine	4	A, D, E, F	[66]
	Vitamin & trace element	Pyridoxine	4	A, D, E, F	[66]
Alpha-fucosidase deficiency	Stem cell therapy	(Umbilical cord/bone marrow) hematopoietic stem cell transplantation	4	D, E, G	[67]
Alpha-iduronidase deficiency (synonyms: Hurler syndrome [severe]; Scheie syndrome [attenuated])	Enzyme replacement therapy	Intrathecal iduronidase	2b	A, D, G	[68, 69]
	Stem cell therapy	Hematopoietic stem cell transplantation	1c	D, G	[68, 69]
Alpha-mannosidase deficiency	Enzyme replacement therapy	Velmanase alfa	1c	D	[70, 71]
	Stem cell therapy	Hematopoietic stem cell transplantation	4 to 5	D, E	[70, 71]
Arginase deficiency (synonym: argininemia)	Nutritional therapy	Protein defined diet, arginine or citrulline	2b, effect on growth 4	B, C, D, E, G	[72]
	Pharmacological therapy	Sodium phenylbutyrate, glycerol phenylbutyrate, sodium benzoate	2b	B, C, D, E	[72]
	Other	Hemodialysis, peritoneal dialysis	4	D	[72]
	Solid organ transplant	Liver transplantation	4	A, B, D, E	[72]
Arginine:glycine amidinotransferase (AGAT) deficiency	Nutritional therapy	Creatine	4	A, D, E	[73]
Argininosuccinate lyase deficiency	Nutritional therapy	Protein defined diet, arginine or citrulline	2b, effect on growth 4	B, C, D, E, G	[72]
	Pharmacological therapy	Sodium phenylbutyrate, glycerol phenylbutyrate, sodium benzoate	2b	B, C, D, E	[72]
	Other	Hemodialysis, peritoneal dialysis	4	D	[72]
	Solid organ transplant	Liver transplantation	4	A, B, D, E, systemic phenotype persists	[72]
Argininosuccinate synthetase deficiency (synonym: citrullinemia type 1)	Nutritional therapy	Protein defined diet, arginine or citrulline	2b, effect on growth 4	B, C, D, E, G	[72]
	Pharmacological therapy	Sodium phenylbutyrate, glycerol phenylbutyrate, sodium benzoate	2b	B, C, D, E	[72]
	Other	Hemodialysis, peritoneal dialysis	4	D	[72]
	Solid organ transplant	Liver transplantation	4	A, B, D, E	[72]
Aromatic L-amino acid decarboxylase deficiency	Pharmacological therapy	Dopamine agonist, monoamine oxidase (MAO) inhibitors, L-dopa + carbidopa (depending on mutation)	4	E, G	[74, 75]
	Vitamin & trace element	Pyridoxine, folinic acid ±	4	E, G	[74, 75]
	Gene-based therapy	Gene therapy	4	A, B, E	[74, 75]
Arylsulfatase A deficiency	Gene-based therapy	OTL-200 (stem cell-based gene therapy)	2c	A, E	[76, 77]
	Stem cell therapy	Hematopoietic stem cell transplantation	4 to 5	D	[76, 77]
Aspartylglucosaminidase deficiency	Stem cell therapy	Hematopoietic stem cell transplantation	4 to 5	D	[78]
ATP-sensitive potassium channel regulatory/pore-forming subunit superactivity	Pharmacological therapy	Sulfonylurea	4	A, B, E, G	[79, 80]
Autosomal recessive GTP cyclohydrolase 1 deficiency	Nutritional therapy	Phenylalanine-reduced diet ±	4	D, E	[62]
	Vitamin & trace element	Folinic acid ±	4	A, E, F	[62]
	Pharmacological therapy	L-dopa + carbidopa, 5-Hydroxytryptophan, sapropterin dihydrochloride (synthetic BH4) co-factor therapy	4, 4 to 5 for 5-Hydroxytryptophan	A, B, E, F, G	[62]
Beta-glucuronidase deficiency (synonym: Sly syndrome)	Enzyme replacement therapy	Vestronidase	4	A, G	[81]
	Stem cell therapy	Hematopoietic stem cell transplantation	4	D, G	
Biotinidase deficiency	Vitamin & trace element	Biotin	2c	A, E, G	[82]
Branched-chain ketoacid dehydrogenase E1 alpha (BCKDHA)/beta (BCKDHB)/E2 (DBT) deficiency (synonym: maple syrup urine disease type 1a (BCKDHA)/2 (DBT); Dihydrolipoyl transacylase deficiency (DBT))	Nutritional therapy	Restriction of branched chain amino acids, isoleucine, valine, avoid fasting, sick day management	4	A, C, D, G	[83, 84]
	Vitamin & trace element	Thiamine ±	4	C, D, G	[83, 84]
	Other	Hemodialysis, peritoneal dialysis	4	D	[83, 84]
	Solid organ transplant	Liver transplantation	4	C, D, G	[83, 84]
Branched-chain ketoacid dehydrogenase kinase deficiency	Nutritional therapy	Branched-chain amino acid supplementation	5	A, B, G	[85]
CAD trifunctional protein deficiency	Pharmacological therapy	Uridine	4	A, F	[30]
Carbamoyl phosphate synthetase 1 deficiency	Nutritional therapy	Protein defined diet, arginine or citrulline	2b, effect on growth 4	B, C, D, E, G	[72]
	Pharmacological therapy	Sodium phenylbutyrate, glycerol phenylbutyrate, sodium benzoate, N-carbamyl-L-glutamate (carglumic acid)	2b, 4 to 5 for carglumic acid	B, C, D, E, D for carglumic acid	[72]
	Other	Hemodialysis, peritoneal dialysis	4	D	[72]
	Solid organ transplant	Liver transplantation	4	A, B, D, E	[72]
Carbonic anhydrase VA deficiency	Nutritional therapy	Sick day management	4	C, D	[86]
	Pharmacological therapy	N-carbamyl-L-glutamate (carglumic acid)	4	C, D	[86]
Citrin deficiency	Nutritional therapy	High-protein/high-fat/low-carbohydrate diet, avoid fasting, avoid glucose iv, lactose restriction, medium -chain triglycerides	4	B, C, D, E, F, G	[87]
	Other	Hemodialysis, peritoneal dialysis	4	D	[87]
	Solid organ transplant	Liver Transplantation	4	C	[87]
CLN7 disease	Gene based therapy	(Intrathecal) milasen	4 to 5	F	[36]
Coenzyme Q5 methyltransferase deficiency	Vitamin & trace element	CoQ10	4 to 5	A, B, E	[88]
Coenzyme Q8A (ADCK3) deficiency	Vitamin & trace element	CoQ10	4 to 5	E	[89]
Copper-transporting ATPase subunit alpha deficiency (synonyms: Menkes disease [severe]; occipital horn syndrome [milder])	Pharmacological therapy	Copper histidine	4	D	[90]
Copper-transporting ATPase subunit beta deficiency (synonym: Wilson disease)	Vitamin & trace element	Zinc	4	A, E, G	[91, 92]
	Pharmacological therapy	Copper chelators, tetrathiomolybdate	4, 1b for tetrathiomolybdate	A, E, G	[91, 92]
Creatine transporter deficiency	Nutritional therapy	Creatine, glycine, arginine	4	A, F	[93]
Cyclic pyranopterin monophosphate synthase deficiency (synonym: molybdenum cofactor deficiency type A)	Pharmacological therapy	Cyclic pyranopterin monophosphate	2b	A, F	[94]
Cystathionine beta-synthase deficiency (synonym: classic homocystinuria)	Nutritional therapy	Protein defined diet, methionine restriction	2c	C, D, G	[95]
	Vitamin & trace element	Pyridoxine, betaine	2c	D, E, G	[95]
Dihydrofolate reductase deficiency	Vitamin & trace element	Folic acid	4	F, G	[96]
Dihydropteridine reductase deficiency	Nutritional therapy	Phenylalanine-reduced diet	4	D, E	[62]
	Vitamin & trace element	Folinic acid	4	A, E, F	[62]
	Pharmacological therapy	L-dopa + carbidopa, 5-Hydroxytryptophan, sapropterin dihydrochloride (synthetic BH4) co-factor therapy ±	4	A, B, E, F, G	[62]
DNAJC12 deficiency	Pharmacological therapy	BH4, L-dopa + carbidopa ± , 5-hydroxytryptophan ±	4	A, D, E	[97]
Electron transfer flavoprotein subunit alpha (ETFA)/subunit beta (ETFB)/dehydrogenase deficiency (ETFDH) (synonym: glutaric acidemia type 2A/2B/2C; multiple acyl-CoA dehydrogenase deficiency type 2A/2B/2C)	Nutritional therapy	Carnitine	5	C, D	[98]
	Vitamin & trace element	Riboflavin	5	C, D	[98]
	Nutritional therapy	Beta-hydroxybutyrate	2c	E, G	[98]
Epsilon-N-trimethyllysine hydroxylase deficiency	Nutritional therapy	Carnitine	4 to 5	A, B, C	[99]
Folate receptor alpha deficiency (synonym: neurodegeneration due to cerebral folate transport deficiency)	Vitamin & trace element	Folinic acid	4	A, D, E, F	[100]
GLUT1 deficiency	Nutritional therapy	Ketogenic diet	4	F	[101]
	Pharmacological therapy	Triheptanoin	2b	A, E	[102]
Glutamine synthetase deficiency	Nutritional therapy	Glutamine	4 to 5	A, E	[103]
Glutaryl-CoA dehydrogenase deficiency (synonym: glutaric aciduria type 1)	Nutritional therapy	Protein defined diet, lysine restriction, carnitine	2c	C, D, E, G	[44]
Glycine encephalopathy due to aminomethyltransferase (AMT) or glycine decarboxylase (GLDC) deficiency (synonym: nonketotic hyperglycinemia)	Pharmacological therapy	Sodium benzoate, N-nitrosodimethylamine receptor antagonists	4	B, D, E, F	[104]
Guanidinoacetate methyltransferase deficiency	Nutritional therapy	Arginine restriction, creatine and ornithine	4	B, D, E, F	[105]
Hereditary ceruloplasmin deficiency	Other	Iron chelation	4	D, E	[106]
Holocarboxylase synthetase deficiency	Vitamin & trace element	Biotin	4	D	[107]
Homocystinuria, cblDv1 type	Vitamin & trace element	Hydroxycobalamin, betaine ±	4	C, D, G	[61]
Iduronate sulfatase deficiency (synonym: Hunter syndrome)	Stem cell therapy	Hematopoietic stem cell transplantation	4	D, E, G	[108]
Ionotropic glutamate receptor NMDA type subunit 1 dysregulation	Pharmacological therapy	Memantine	4 to 5	A, F	[109]
Ionotropic glutamate receptor NMDA type subunit 2A dysregulation	Pharmacological therapy	Memantine, IVIG	4	F	[110, 111]
Ionotropic glutamate receptor NMDA type subunit 2B dysregulation	Nutritional therapy	L-serine (for loss-of-function mutations)	4 to 5	A	[112]
Ionotropic glutamate receptor NMDA type subunit 2D superactivity	Pharmacological therapy	Memantine, IVIG	4	A, F	[111, 113]
Isoleucyl-tRNA synthetase 1 deficiency	Nutritional therapy	L-isoleucine, natural protein fortification	4 to 5	A, E, G	[114, 115]
Isovaleryl-CoA dehydrogenase deficiency (synonym: isovaleric acidemia)	Nutritional therapy	Protein defined diet, carnitine, avoid fasting, sick day management	2c	C, G	[116]
	Pharmacological therapy	N-carbamyl-L-glutamate (carglumic acid)	4	C, G	[116]
Leucyl-tRNA synthetase 1 deficiency	Nutritional therapy	L-leucine, natural protein fortification	4 to 5	A, E, G	[114, 115]
MEDNIK syndrome	Vitamin & trace element	Zinc acetate	4 to 5	A, B, G	[117]
Methionine synthase deficiency (synonym: homocystinuria-megaloblastic anemia, cblG type)	Vitamin & trace element	Hydroxycobalamin, betaine ±	4, 5 for betaine	C, D, G	[61]
Methionine synthase reductase deficiency (synonym: homocystinuria-megaloblastic anemia, cblE type)	Vitamin & trace element	Hydroxycobalamin, betaine ±	4, 5 for betaine	C, D, G	[61]
Methionyl-tRNA synthetase 1 deficiency	Nutritional therapy	Methionine, increase protein intake	5	A, G	[118]
Methylmalonic aciduria and homocystinuria, cblC type	Nutritional therapy	Carnitine ±	5	C, D, G	[61]
	Vitamin & trace element	Hydroxycobalamin, betaine	4	C, D, G	[61]
Methylmalonic aciduria and homocystinuria, cblD type	Nutritional therapy	Carnitine ±	5	A, D, G	[61]
	Vitamin & trace element	Hydroxycobalamin, betaine ±	4 to 5, 5 for betaine	A, D, G	[61]
Methylmalonic aciduria and homocystinuria, cblF type	Vitamin & trace element	Hydroxycobalamin, betaine	4	D, G	[61]
Methylmalonic aciduria and homocystinuria, cblJ type	Vitamin & trace element	Hydroxycobalamin, betaine ±	4 to 5, 5 for betaine	D, G	[119]
Methylmalonic aciduria due to methylmalonyl-CoA mutase deficiency	Nutritional therapy	Protein defined diet, carnitine, avoid fasting, sick day management	2c	C, D, G	[120]
	Pharmacological therapy	N-carbamyl-L-glutamate (carglumic acid), sodium benzoate, antibiotics	4	C, D, G	[120]
	Other	Hemodialysis, peritoneal dialysis	4	D	[120]
	Solid organ transplant	Liver and/or kidney transplantation	4	C, D, G	[120]
Methylmalonic aciduria, cblA type	Nutritional therapy	Protein defined diet, carnitine, avoid fasting, sick day management	4	C, D, G	[120]
	Vitamin & trace element	Hydroxycobalamin	4	C, D, G	[120]
	Pharmacological therapy	N-carbamyl-L-glutamate (carglumic acid), sodium benzoate, antibiotics	4	D	[120]
	Other	Hemodialysis, peritoneal dialysis	4	D	[120]
	Solid organ transplant	Liver transplantation and/or kidney transplantation	4	C, D, G	[120]
Methylmalonic aciduria, cblB type	Nutritional therapy	Protein defined diet, carnitine, avoid fasting, sick day management	4	C, D, G	[120]
	Vitamin & trace element	Hydroxycobalamin	4	C, D, G	[120]
	Pharmacological therapy	N-carbamyl-L-glutamate (carglumic acid), sodium benzoate, antibiotics	4	D	[120]
	Other	Hemodialysis, peritoneal dialysis	4	D	[120]
	Solid organ transplant	Liver transplantation and/or kidney transplantation	4	C, D, G	[120]
Methylmalonic aciduria, cblDv2 type	Nutritional therapy	Protein defined diet, carnitine, avoid fasting, sick day management	4	C, D, G	[120]
	Vitamin & trace element	Hydroxycobalamin	4	C, D, G	[120]
	Pharmacological therapy	N-carbamyl-L-glutamate (carglumic acid), sodium benzoate, antibiotics	4	D	[120]
	Other	Hemodialysis, peritoneal dialysis	4	D	[120]
	Solid organ transplant	Liver transplantation and/or kidney transplantation	4	C, D, G	[120]
Mitochondrial 3-hydroxy-3-methylglutaryl-CoA synthase deficiency	Nutritional therapy	Avoid Fasting, sick day management, dietary precursor restriction ±	5	C	[121]
Mitochondrial acetoacetyl-CoA thiolase deficiency	Nutritional therapy	Avoid fasting, sick day management, protein restriction, isoleucine restriction	5	C	[122]
Mitochondrial aspartate aminotransferase deficiency	Nutritional therapy	L-serine	4	A	[123]
	Vitamin & trace element	Pyridoxine	4	A, F	[123]
Mitochondrial aspartate-glutamate carrier isoform 1 deficiency (synonym: aralar deficiency)	Nutritional therapy	Ketogenic diet	4	A, E, F	[124]
Mitochondrial myopathy, encephalopathy, lactic acidosis and stroke-like episodes (MT-ND1/MT-ND4/MT-ND5/MT-ND6/MT-CO1/MT-CPO2/MT-CO3/ MT-TQ/MT-TH/MT-TL1/MT-TF/MT-TS1/MT-TS2/MT-TW)	Nutritional therapy	Arginine, citrulline, taurine	4 to 5, 2b for Taurine	C, D, E, F	[125, 126]
Mitochondrial ornithine transporter deficiency (synonym: hyperornithinemia-hyperammonemia-homocitrullinuria syndrome)	Nutritional therapy	Protein defined diet, arginine or citrulline	2b, effect on growth 4	B, C, D, E, G	[72]
	Pharmacological therapy	Sodium phenylbutyrate, glycerol phenylbutyrate, sodium benzoate	2b	B, C, D, E	[72]
	Other	Hemodialysis, peritoneal dialysis	4	D	[72]
	Solid organ transplant	Liver transplantation	4 to 5	A, B, D, E, systemic phenotype persists	[72]
Mitochondrial short-chain enoyl-CoA hydratase 1 deficiency	Nutritional therapy	Valine restriction	4	A, E, G	[58]
Mitochondrial sulfur dioxygenase deficiency (synonym: ethylmalonic encephalopathy)	Pharmacological therapy	N-acetylcysteine, antibiotics	4	E, G	[127]
	Solid organ transplant	Liver transplantation	4	A	[127]
Mitochondrial thiamine pyrophosphate transporter deficiency	Vitamin & trace element	Thiamine	4	C, D	[128]
N-acetylglutamate synthase deficiency	Nutritional therapy	Protein defined diet, arginine or citrulline	2b, effect on growth 4	B, C, D, E, G	[72]
	Pharmacological therapy	Sodium phenylbutyrate, glycerol phenylbutyrate, sodium benzoate, N-carbamyl-L-glutamate (carglumic acid)	2b, 4 for carglumic acid	B, C, D, E, D for carglumic acid	[72]
	Other	Hemodialysis, peritoneal dialysis	4	D	[72]
	Solid organ transplant	Liver transplantation	4	A, B, D, E	[72]
NAD(P)HX epimerase deficiency	Vitamin & trace element	Niacin, CoQ10	4 to 5	A, G	[129]
Niemann-Pick disease type C1 (NPC1)/C2 (NPC2)	Pharmacological therapy	Miglustat, intrathecal 2-hydroxypropyl-β-cyclodextrin	1b, 2b for 2-hydroxypropyl-β-cyclodextrin	D, E	[130, 131]
NRF2 superactivity (synonym: immunodeficiency, developmental delay, and hypohomocysteinemia)	Vitamin & trace element	Ascorbic acid	4 to 5	A	[132]
	Pharmacological therapy	Luteolin	4 to 5	A	[132]
Ornithine transcarbamylase deficiency	Nutritional therapy	Protein defined diet, arginine or citrulline	2b, effect on growth 4	B, C, D, E, G	[72]
	Pharmacological therapy	Sodium phenylbutyrate, glycerol phenylbutyrate, sodium benzoate	2b	B, C, D, E	[72]
	Other	Hemodialysis, peritoneal dialysis	4	D	[72]
	Solid organ transplant	Liver transplantation	4	A, B, D, E	[72]
Phenylalanine hydroxylase deficiency (synonym: phenylketonuria)	Nutritional therapy	Protein defined diet, phenylalanine-free L-amino acid suppletion/Glycomacropeptide (GMP), large neutral amino acid (LNAA), fatty acids	2a, GMP 4	B, D, E	[43]
	Pharmacological therapy	Sapropterin dihydrochloride (synthetic BH4) co-factor therapy	1b	B, D, E	[43]
	Enzyme replacement therapy	Pegvaliase	1b	B, D, E	[43]
Phenylalanyl-tRNA synthetase subunit beta deficiency	Nutritional therapy	L-phenylalanine	4 to 5	A, G	[115]
Phosphoribosylpyrophosphate synthetase deficiency	Pharmacological therapy	S-adenosylmethionine	4	D, G	[133]
Phosphoserine aminotransferase deficiency	Nutritional therapy	L-Serine, glycine	4	D, E, F	[59]
Phosphoserine phosphatase deficiency	Nutritional therapy	L-Serine, glycine	4	D, E, F	[59]
PIGA-CDG	Nutritional therapy	Ketogenic diet	4	A, F	[134]
PIGM-CDG	Pharmacological therapy	Sodium phenylbutyrate	4	A, F	[134]
PIGO-CDG	Vitamin & trace element	Pyridoxine	4 to 5	F	[134]
PMM2-CDG	Pharmacological therapy	Acetazolamide	1b	E, G	[135]
Propionic acidemia due to propionyl-CoA carboxylase subunit alpha (PCCA)/beta (PCCB) deficiency	Nutritional therapy	Protein defined diet, carnitine, avoid fasting, sick day management	2c	C, D, G	[120]
	Pharmacological therapy	N-carbamyl-L-glutamate (carglumic acid), sodium benzoate, antibiotics	4	C, D, G	[120]
	Other	Hemodialysis, peritoneal dialysis	4	D	[120]
	Solid organ transplant	Liver and/or kidney transplantation	4	C, D, G	[120]
Proton-coupled folate transporter deficiency (synonym: hereditary folate malabsorption)	Vitamin & trace element	(Levo-)folinic acid	4	A, E, F, G	[136]
Pyridoxamine 5′-phosphate oxidase deficiency	Vitamin & trace element	Pyridoxal phosphate	4	D, E, F	[137]
Pyruvate dehydrogenase E1 alpha (PDHA1)/beta (PDHB)/E2 (DLAT)/E3 (DLD)/E3BP (PDHX) deficiency	Nutritional therapy	Ketogenic diet	4 for PDHA1 and PDHX, 5 for other genes	A, D, E, F	[138, 139]
	Vitamin & trace element	Thiamine	4 for some mutations	D, E, F	[138, 139]
Pyruvate dehydrogenase phosphatase deficiency	Nutritional therapy	Ketogenic diet, thiamine ±	4	A, D, E	[140]
S-adenosylhomocysteine hydrolase deficiency	Nutritional therapy	Methionine restriction, creatinine, phosphatidylcholine	4	A, E, G	[141]
	Solid organ transplant	Liver transplantation	4 to 5	A, E, G	[141]
Sepiapterin reductase deficiency	Pharmacological therapy	L-dopa + carbidopa, 5-Hydroxytryptophan	4	A, B, E, F, G	[62]
	Vitamin & trace element	Folinic acid ±	5	A, E, F	[62]
Seryl-tRNA synthetase 1 deficiency	Nutritional therapy	L-serine	4 to 5	A, B, G	[114, 115]
SLC35A2-CDG	Nutritional therapy	Galactose	4	E, F, G	[142]
SLC35C1-CDG	Nutritional therapy	Fucose	4	A, G	[134]
SLC39A8 deficiency	Nutritional therapy	Galactose, manganese, uridine	4	F	[134]
Sodium-dependent multivitamin transporter deficiency	Vitamin & trace element	Biotin, alpha-lipoic acid, pantothenic acid	4	A, D, G	[143]
Sterol 27-hydroxylase deficiency (synonym: cerebrotendinous xanthomatosis)	Pharmacological therapy	Chenodeoxycholic Acid	3a	B, D, E, G	[144]
Succinic semialdehyde dehydrogenase deficiency	Pharmacological therapy	Vigabatrin	4	B, F	[145]
Thiamine pyrophosphokinase deficiency	Vitamin & trace element	Thiamine	4	D	[146]
Thiamine transporter 2 deficiency (synonym: biotin-thiamine-responsive basal ganglia disease)	Vitamin & trace element	Thiamine, biotin	2c for thiamine, 4 for biotin	D	[147]
Transcobalamin II deficiency	Vitamin & trace element	Cyanocobalamin, hydroxycobalamin	4	D, E, G	[148]
Tripeptidyl-peptidase 1 deficiency (synonym: CLN2 disease)	Enzyme replacement therapy	Cerliponase alfa	2b	D	[149]
Tyrosine aminotransferase deficiency (synonyms: tyrosinemia type 2; Richner-Hanhart syndrome)	Nutritional therapy	Protein defined diet, phenylalanine and tyrosine restriction	4 G, 5 for D	D, G	[150]
Tyrosine hydroxylase deficiency	Pharmacological therapy	L-dopa + carbidopa	4	A, E	[151]
Vesicular monoamine transporter 2 deficiency	Pharmacological therapy	Pramipexol (dopamine agonist)	4	A, E	[152]
X-linked adrenoleukodystrophy	Gene-based therapy	Gene therapy	5	D, E	[153]
	Stem cell therapy	Hematopoietic stem cell transplantation	1c	D, E	[154]

Level of evidence: Level 1a = systematic review of RCT's, 1b = individual RCT, 1c = ‘All or None’ [= (prolongation of) survival with therapy]; Level 2a = systematic review of cohort studies, 2b = individual cohort study, 2c = ‘Outcomes Research’ [focused on end results of therapy for chronic conditions, including functioning and quality of life]; Level 3 = systematic review of case– control studies; Level 4 = individual case–control study or case-series/report; Level 5 = expert opinion without critical appraisal; based on physiology, bench research or first principles. If only one patient was reported, we assigned level ‘4 to 5′ as a way to nuance the treatment effects

Effect of treatment: A = improves psychomotor/cognitive development/IQ, B = improves behavioural/psychiatric disturbance(s), C = prevents acute metabolic decompensation, D = prevents, halts, or slows clinical deterioration, E = improves neurological manifestations (incl. neuro-imaging), F = improves seizure/epilepsy control, G = improves systemic manifestations

#### Classification

Included treatable IDs belonged to the following 17 ICIMD categories: vitamin and cofactor metabolism 29 (25%), amino acid metabolism 28 (24%), complex molecule degradation 10 (9%), neurotransmitters 9 (8%), nucleobase, nucleotide and nucleic acid metabolism 7 (6%), disorders of glycosylation 6 (5%), energy substrate metabolism 5 (4%), trace elements and metals 5 (4%), fatty acid, carnitine, and ketone body metabolism 5 (4%), lipid metabolism 3 (3%), mitochondrial cofactor biosynthesis 2 (2%), other disorders of mitochondrial function 2 (2%), carbohydrate metabolism 1 (1%), peptide and amine metabolism 1 (1%), endocrine metabolic disorders 1 (1%), mtDNA-related disorders 1 (1%), and nuclear-encoded disorders of oxidative phosphorylation 1 (1%).

#### Types of treatment and levels of evidence

The different types of treatment as defined in Table 1A are shown per disorder in Table 3. Nutritional therapy was the most frequently used treatment strategy (32%), followed by pharmacological therapy 22%, vitamin and trace element substitution 22%, solid organ transplantation 8%, hematopoietic stem cell transplant 4%, enzyme replacement therapy 3%, gene-based therapy 2% and other therapy 7% (multiple treatments per disease entity were possible).

The level of evidence for each treatment as defined in Table 1A is shown in Table 3. Most often, case series or case reports with an evidence level of 4 (60%), 4–5 (12%) and 5 (8%) were reported; higher evidence levels accounted for a minority (level 1 for 4%, level 2 for 14%, level 3 for 0.5%) (Table 3).

#### Effect(s) of treatments on outcome measures

Treatment prevented, halted, or slowed clinical deterioration in 62%, improved neurological manifestations (incl. neuro-imaging) in 47%, systemic manifestations in 44% and psychomotor/cognitive development/IQ in 37%; it prevented acute metabolic decompensation in 30%, improved seizure/epilepsy control in 22% and improved behavioural/psychiatric disturbance(s) in 21%.

### Diagnostic algorithm

**Fig. 1:**
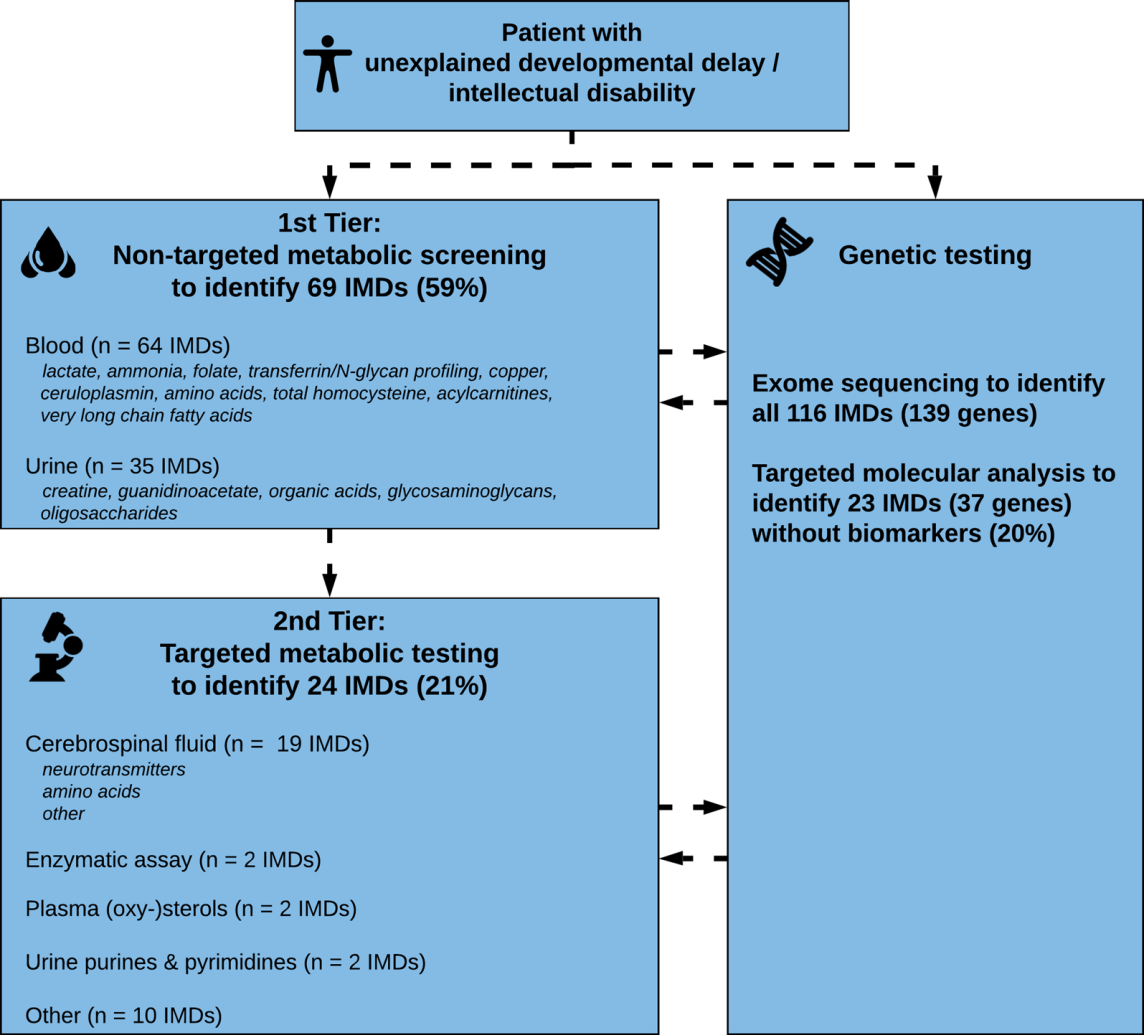
Diagnostic algorithm for treatable IDs. 1st Tier consists of non-targeted metabolic screening tests that are readily available in most developed countries. 2nd Tier consists of targeted metabolic tests, often more invasive and/or less available. Some IMDs are identified by multiple (screening) tests in the 1st and 2nd Tier. Genetic testing (targeted molecular analysis as well as exome sequencing) can be performed in parallel. (ID = Intellectual disability; IMD = Inherited Metabolic Disorder)

The diagnostic algorithm in Fig. 1 is proposed for the evaluation of a patient of any age presenting with DD or ID in whom the cause is not apparent. It is based on the following results: 1^st^ Tier or ‘basic’ metabolic screening (in blood: lactate, plasma ammonia, serum transferrin/N-glycan profiling, serum/red blood cell folate, serum copper and ceruloplasmin, plasma amino acids, plasma total homocysteine, plasma (or dried bloodspot) acylcarnitines, very long chain fatty acids; in urine: organic acids, creatine, guanidinoacetate, glycosaminoglycans and oligosaccharides) is available at most if not all diagnostic metabolic laboratories and can identify 69 (59%) of the 116 treatable IDs. Of course, further biochemical and genetic confirmation is warranted, but for the sake of prompt initiation of treatment, these first-tier results yield sufficient diagnostic information.

Importantly, for 23 of 116 IMDs (20%) no specific biomarker is currently available and thus molecular testing (targeted or via exome) is required (Fig. 1).

Lumbar puncture for cerebrospinal fluid (CSF) neurotransmitter and amino-acid analysis is indicated in patients with neonatal or infantile seizures of unknown aetiology, dystonia or other movement disorders, progressive intellectual neurological deterioration (85 IMDs, 41% amenable to treatment [26]), severe psychiatric or behavioral phenotypes, and/or clinical findings suggestive of dopamine deficiency (hypersalivation, temperature dysregulation, oculogyric crises, or hypokinesia). An abnormal profile has important diagnostic as well as therapeutic implications, such as neurotransmitter supplementation, e.g., in GTCPH deficiency, or even in additional non-classic IMDs, e.g., in a PAK3 deficiency patient with severe automutilation and low CSF homovanillic acid (HVA) [27].

### Treatable ID App

**Fig. 2:**
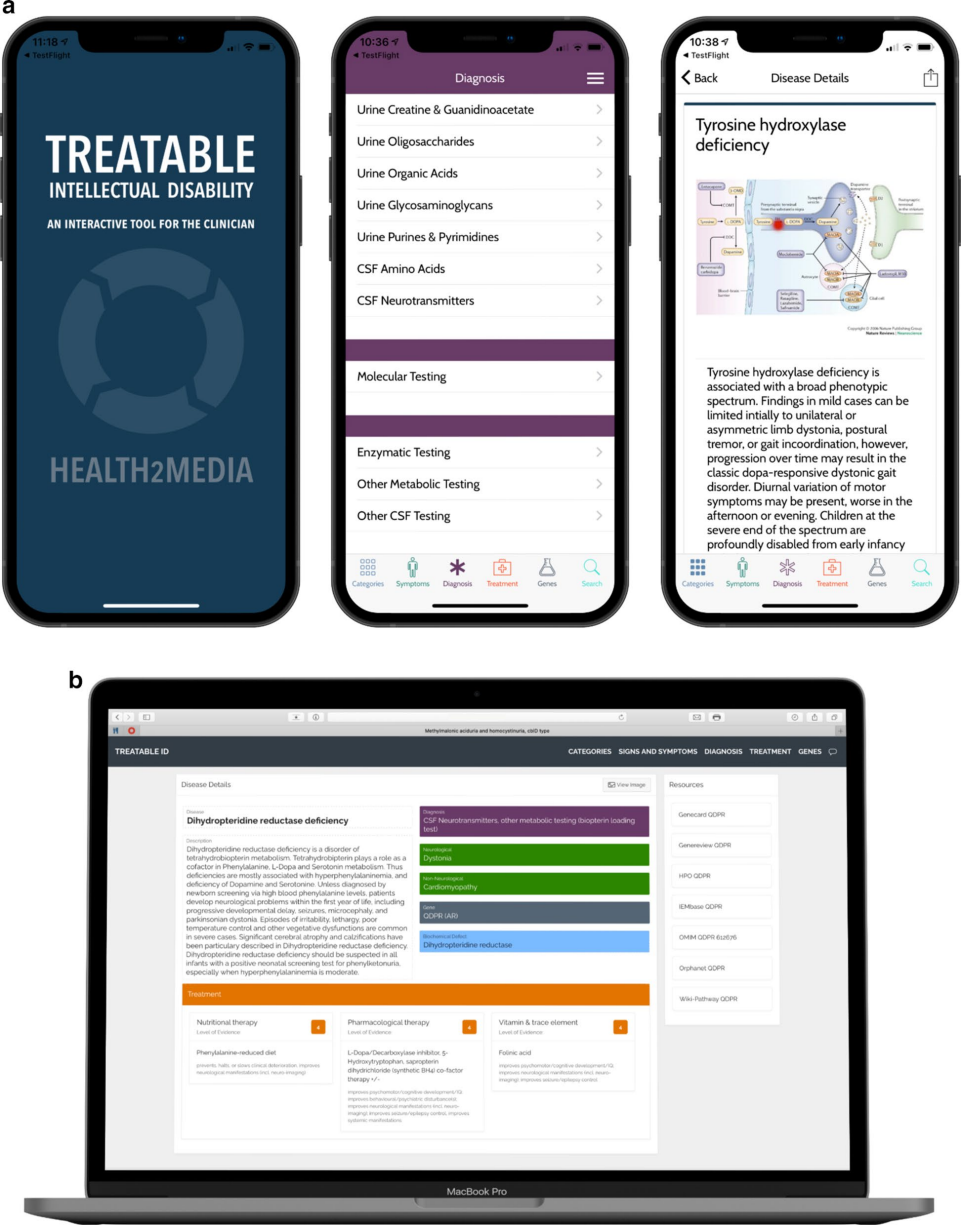
The updated Treatable ID App, an interactive digital tool for the clinician to **a** search for IMDs according to genes, signs and symptoms, diagnostic tests, and treatments, and **b** find information on specific IMDs along with links to other digital resources. (IMD = Inherited Metabolic Disorder)

First, based on our 2021 literature search, the content has been updated in the Treatable ID App, including updated summaries, symptoms, diagnostic tests, treatments with levels of evidence and effects for each IMD. A disorder page has been designed of each IMD with links to Orphanet, OMIM, Human Phenotype Ontology (HPO), GeneReviews, GeneCards, IMDbase, Vademecum Metabolicum and WikiPathways. Second, based on user feedback, the design has been updated with a quick and solid interface. It is possible to search based on signs and symptoms for IMDs, the appropriate diagnostic tests and gene lists and panel designs, available treatments and evidence. Figure 2 illustrates the Treatable ID App features on mobile devices. The App is available via the App Store, Google Play and online (http://www.treatable-id.org) freely downloadable for all interested users including but not limited to general practitioners, medical and biochemical geneticists, neurologists, developmental and pediatricians, internists, metabolic diseases specialists, as well as laboratory specialists and patients and families.

## Discussion

### Targeted therapies for treatable IDs: strengthening the Treatabolome

Our systematic review from 2012 was the first to prioritize the treatability in the diagnostic evaluation of patients with DD and ID in whom the cause was not evident after a thorough clinical exam. As increasing numbers of patients are diagnosed through exome and genome sequencing in clinical practice or within research projects, it becomes ever more crucial to enable flagging of potentially treatable cases at a gene or variant level. However, this knowledge was (and still is) largely available only in ‘human-readable’ scientific publications or in expert practice, and our website and app were the first to capture this knowledge in a computer-accessible form that would allow automatic recognition and flagging in analysis and decision-support systems. The implementation of these web resources in national guidelines as well as treatment programs has successfully changed the clinical practice and inspired several reviews on other genetic diseases (e.g., neuromuscular disease [28], epilepsy [17] or movement disorders [29]). The final goal is a ‘treatabolome’ database accessible for all health care providers as well as patients.

### Diagnostics advances

The field of IMDs is a moving target with 44 newly-defined IMDs included in our recent update, and still the IMDs are the largest group of monogenic conditions underlying ID amenable to treatment. The wide implementation of NGS techniques has led to the identification of new IMDs and the better understanding of the underlying pathophysiology has led to a substantial change in the definition of IMDs (‘Any condition in which the impairment of a biochemical pathway is intrinsic to the pathophysiology of the disease, regardless of whether there are abnormalities in currently available biochemical laboratory tests’). These changes led to an increasing number of individuals being diagnosed with IMDs for which no metabolic marker is available (e.g., CAD deficiency [30]), in turn leading to prioritization of exome sequencing (panels) in the diagnostic algorithm. However, these techniques are not always available or have significantly longer turnaround times than conventional metabolic screening in body fluids. Conversely, about 50% of all individuals undergoing genetic testing for ID remain without a diagnosis, which still makes metabolic screening an important part of the diagnostic workup. This is further underlined by a recent study on genomic newborn screening, showing that ES identifies only 9 out of 10 IMDs picked up reliably by tandem mass spectrometry. Thus picking up all patients eligible for early intervention urges a combined approach [31] The 1st and 2nd tier metabolic tests will also identify IDs not amenable to targeted therapy. Of course, any type of diagnosis is beneficial as it provides closure, end to a diagnostic odyssey, information to patients and families, as well as access to accurate genetic counseling, supportive care and reimbursement.

The exact order of diagnostic tests still depends on local resources and expertise and needs critical appraisal and personalization of the subsequent treatment itself. The diagnostic algorithm as shown in Fig. 1 is our recommendation, based on the yield of metabolic tests combined in the tiers, as well as availability at most if not all metabolic laboratories. The algorithm can be adapted according to the clinician’s insights and laboratory specialist’s expertise, as well as the patient’s clinical phenotype (red flags) along with local laboratory resources.

Increasingly, metabolomics or next generation metabolomic screening (NGMS) will replace the individual biochemical tests and assays, similar to ES replacing single gene tests or panels [32]. Already today, most genetic laboratories solely work with ES and use virtual panels or virtual single gene analysis when these tests are required. Hence, in most clinical situations, it seems reasonable to initiate metabolic screening and genetic testing in parallel. The metabolic screening results can validate the functional impact of genetic variants identified by ES (eg N-acetyl-mannosamine for NANS-CDG [33]), thus providing a functional read-out. In ES negative patients, metabolic aberrations can help to guide the genetic investigations to scrutinize genes in specific pathways and vice versa, the genetic results can direct the NGMS interpretation and/or require additional specific metabolic testing (e.g., enzyme assays). In life-threatening situations or Progressive Intellectual and Neurologic Deterioration (PIND), quick turn-around is indicated for both metabolic/NGMS screening (within hours) and ‘accelerated’/ ‘Turbo’ genome sequencing whenever available.

### Treatment advances

In parallel, rare disease and (tailored) genetic therapies are gaining mainstream attention with the Food and Drug Administration (FDA) approval of the antisense oligonucleotide (Nusinersen) as well as the gene therapy (onasemnogene abeparvovec) for Spinal muscular atrophy (SMA) [34, 35] and the first personalized antisense oligonucleotide (Milasen) for CLN7-related neurodegeneration [36].

Our data show that although there has been much attention given to gene-based and enzyme replacement therapy, the majority of currently available, effective treatment strategies are nutritional, via dietary interventions and supplementation of vitamins and trace elements. These are relatively cheap, widely available, non-invasive and can be surprisingly effective.

Outcomes of disease and effect of treatment vary widely, depending on the IMD and therapy in question as well as the severity of the phenotype, the disease course and phase, the patient’s age and co-morbidity as well as yet unknown factors. For some IMDs, timely and continued treatment ensures patients live (almost) normal lives, with Phenylketonuria (PKU) being the most prominent example. PKU is not only a superb example for a treatable ID managed with a ‘classical’ nutritional intervention to restrict the toxic substrate, but also how further research can broaden the therapeutic opportunities (e.g., tetrahydrobiopterin, Pegvaliase) and how the genotype can help in treatment planning.

We decided to exclude certain disorders where ID is no longer considered one of the main clinical features (e.g., Succinyl-CoA:3-oxoacid-CoA transferase (SCOT) deficiency, Riboflavin transporter deficiency (RTD)), or where treatment was shown to be ineffective in larger studies (e.g., Sanfilippo syndrome A-D). However, with the increasing focus on personalized treatment we realize that some therapies will show positive effects in selected individuals, while not meeting the “evidence-based” definition for group as a whole.

As our literature review shows, new disorders have been added to this list at a high pace, with 44 new disorders added since 2014. Representative examples include CAD trifunctional protein deficiency in which oral supplementation of uridine (monophosphate) has shown to dramatically improve epilepsy and enable psychomotor development [30, 37]; or memantine repurposing for the treatment of Ionotropic glutamate receptor NMDA type subunit 2A (GRIN2A) dysregulation. For other IMDs, even with treatment, patients still have very severe symptoms, with improvement of IQ seldom reported, but often with improvement of communication skills and behavior. Also, improvement of seizure frequency and intensity can be achieved. While data are scarce to prove the assumption that early treatment will lead to better outcomes, this still seems logical and should prompt the earliest possible diagnosis.

### Prioritizing treatable IMDs in the ID workup

The systematic review of 2012 and its update in 2014 were the first evidence-based approach to demonstrate the significance of IMDs in the diagnostic work up of ID/DD. Whilst most recommendations for the diagnostic workup of DD/ID prioritize frequency of conditions and yield of diagnostic tests, our approach prioritized treatability over frequency and strategizes metabolic/biochemical evaluation in a two-tiered fashion. The utility of this approach was recently shown in the 7-year TIDE-BC study, where we used the two-tiered TIDE diagnostic algorithm for treatable IMD detection superimposed onto current guidelines for the evaluation of unexplained ID. A total of 498 patients (63% male) patients were enrolled; etiologic diagnoses were established in 260 patients (52%), including treatable IMDs in 5%, a third of whom presented with nonspecific symptoms not primarily suggestive of an IMD. Another 15 individuals were diagnosed with a non-treatable IEM [16]. This study represents the first comprehensive metabolic evaluation of a large cohort of patients with ID that is broadly representative of the population seen in a tertiary centre with a biochemical and genetic focus, and therefore possible bias towards IMD. The 29 treatable IMD cases identified and the high overall etiologic diagnosis rate demonstrate the clinical utility of overlaying the TIDE protocol onto current guidelines [16]. The results emphasize the importance of testing for treatable IEMs in all patients with unexplained ID, as earlier diagnosis provides the opportunity to mitigate or possibly prevent irreversible brain damage. Even IMDs for which the typical phenotype comprises complex neurologic abnormalities, PIND or multi-organ disease rather than isolated or stable ID, the earliest or presenting symptom is often developmental delay only (e.g. mucopolysaccharidoses) and thus applying the proposed algorithm and App broadly will enhance early pick-up and treatment of IMDs.

### Treatable ID App

The updated Treatable ID App is designed to serve as an up-to-date, easily accessible and freely available digital tool for all clinicians and laboratory specialists evaluating children presenting with ID. Given the challenge of keeping up with clinical and scientific developments, possibly even more so for rare disorders, this app is meant to facilitate this process and translate the vast knowledgebase into a handy resource for early diagnosis and timely treatment. Searching based on signs and symptoms to generate a differential diagnosis and overviews of tests and treatments is a new feature. Also, a gene list for targeted exome analysis can be generated. The App is kept succinct in order to facilitate quick and easy use. Links are provided to more extensive databases, such as IEMbase [8], that provide more details on diagnostics and symptoms. This way, clinicians can easily access more information as needed. Obviously, the Treatable ID App will need to be updated regularly to reflect advances in the field. The current implementation is designed to facilitate this updating process, every 12 months.

### Limitations and improvements

#### Treatobolome database

The development of the ‘treatabolome’ database poses many challenges, especially with regard to curation, and will require expert input from both the clinical domain and the computational and data stewardship domain.

#### Multi-omics to improve metabolic and genetic (newborn) screening

Another ongoing challenge is the improvement of metabolic screening towards comprehensive tests (next generation metabolic screening, combining metabolomics, glycomics and lipidomics, etc.) [32]. This is in line with the needs to improve and extend genetic screening methods by adding mitochondrial DNA analysis from exome data, genome sequencing and RNA sequencing to the ‘first tier’ genetic toolbox [38] [39] [40]. The implementation of genetic screening into newborn screening programs together with metabolic screening is one of the ongoing challenges.

#### Standardizing therapies and creating higher evidence levels

Our literature search revealed several shortcomings (for a general review on this topic, the evidence creation in IMD, see *Stockler-Ipsiroglu *et al. [2]) that need to be addressed in the future. The evidence of the investigated literature is low. Guidelines, following methodologies established by Scottish Intercollegiate Guideline Network (SIGN) [41] and Grading of Recommendations, Assessment, Development and Evaluations (GRADE) [42] based on rigorous evidence rating and transparent grading of recommendations have become an important tool for the standardization of clinical management of IMDs. These are, however, only available for 13 of the > 1400 known IMDs (e.g., for PKU [43], Glutaric aciduria I [44] or Cobalamin deficiencies [45]). This is reflected by our literature review identifying most often non-analytic case series or case reports with an evidence level of 4 (60%), 4–5 (12%) and 5 (8%).

Because of the nature of rare disorders itself and the current trend to simultaneously develop (or better explore) different treatment modalities for single IMDs, alternative clinical trial designs with the ability to evaluate treatments in small populations within a short time are needed more than ever [46].

With the emergence of drug repurposing for personalized therapies, N-of-1 trials will be increasingly used for proof of principle studies in single patients. Such trial designs will address clinical heterogeneity [47], and incorporate personally meaningful outcomes reflecting patients’ preferences and real world daily experience of the rare disease. In cases where clinical trials cannot be performed, registries with well-defined clinical endpoints can elevate the evidence created by registry-based trials. Adopting common data elements with standardized ontologies [48] as well as the agreement on core outcome sets, [49] is now considered a prerequisite for comparability of data collected across the medical systems caring for rare diseases patients.

#### Access of patients to new technologies and treatments

Our Treatable ID App and algorithm aligns with the vision of the International Rare Diseases Research Consortium to enable all people living with a rare disease to receive an accurate diagnosis and personalized care plan. However access to therapy is still a considerable hurdle in many parts of the world. European Reference Networks and designated centres of expertise address this medical gap. To ‘leave no one behind’, we encourage timely refer all diagnosed patients (at least once) to a such centre to enable access to therapeutic interventions as well as participation in clinical trials and other research studies. Expertise in compounding drugs and magistral preparations is essential for equitable access, as well as a voice in health policy on reimbursement of and access to (orphan) drug and nutritional therapy reimbursement [50] [51] [52].

## Conclusions

Treatable IMDs are a moving target. The broad implementation of next-generation genomic and metabolomic testing in daily clinical practice has accelerated the diagnostics for many individuals with ID. In parallel, the increasing knowledge about the genetic basis of disease, insights into pathophysiology, and advances in therapeutic and targeting strategies catalyze the Treatabolome as a whole; this is true as well for IMDs causing ID. At the same time, methods for evidence generation with small patient numbers as well as more extensive and longer-term follow-up studies will reveal that some therapeutic interventions initially deemed effective do not alter primary or secondary outcomes. These exciting developments require continuous updates of the Treatable ID App, the Treatabolome database, as well as other digital resources. In parallel, diagnostic protocols—whether metabolic or genomic—should also be adjusted to prioritize treatable conditions in the diagnostic workup of suspected IMDs and ensure the earliest possible intervention. We encourage clinicians to use our App to facilitate diagnosis and intervention for treatable IDs, and welcome all feedback including treatable IDs we may have missed.

## Supplementary Information

**Additional File 1**. Inherited metabolic disorders (IMDs) included in our 2012 (PMID 22212131) and 2014 (PMID 24518794) reviews and currently excluded.

## Data Availability

All data generated and analyzed during this study are included in this published article, the supplementary information files and the Treatable ID app (www. Treatable-id.org). The literature search and data are available upon request.

## Abbreviations

ES: Exome sequencing
DD: Global developmental delay
IMD: Inherited metabolic disorder
ICIMD: International classification of inherited metabolic disorders
ID: Intellectual disability
CSF: Cerebrospinal fluid
HVA: Homovanillic acid
NGS: Next generation sequencing
NGMS: Next generation metabolomic screening
PIND: Progressive intellectual and neurologic deterioration
SMA: Spinal muscular atrophy
PKU: Phenylketonuria
COS: Core outcome measure set
