# Supplementary material for: Treatable inherited metabolic disorders causing intellectual disability: 2021 review and digital app
Source: Orphanet J Rare Dis. 2021 Apr 12;16:170. doi: 10.1186/s13023-021-01727-2 (PMC8042729; doi:10.1186/s13023-021-01727-2)
Supplement: Supplementary file 1 — Additional File 1. Inherited metabolic disorders (IMDs) included in our 2012 (PMID 22212131) and 2014 (PMID 24518794) reviews and currently excluded. [file 13023_2021_1727_MOESM1_ESM.docx]

**SUPPLEMENT**

**Supplementary table 1**

IMDs included in previous reviews and currently excluded with stated reason.

| Name of disorder | Gene(s) | Orphanet# | OMIM gene/locus# | HPO# | Reason for exclusion |
| --- | --- | --- | --- | --- | --- |
| Congenital intrinsic factor deficiency | *CBLIF (AR)* | 332 | 609342 | 2694 | ID no longer considered a major clinical feature |
| Imerslund-Gräsbeck Syndrome | *CUBN (AR)* | 35858 | 602997 | 8029 | ID no longer considered a major clinical feature |
| 3-Methylcrotonylglycinuria | *MCCC2 (AR)* | 6 | 609014 | 64087 | ID no longer considered a major clinical feature |
| Pterin-4-alpha-carbinolamine dehydratase (PCD) deficiency | *PCBD1 (AR)* | 1578 | 126090 | 5092 | ID no longer considered a major clinical feature |
| Pyrimidine 5’-Nucleotidase superactivity | *NT5C3A (AR)* | 35120 | 606224 | 51251 | ID no longer considered a major clinical feature |
| Succinyl-CoA:3-oxoacid-CoA transferase (SCOT) deficiency | *OXCT1 (AR)* | 832 | 601424 | 5019 | ID no longer considered a major clinical feature |
| Riboflavin transporter deficiency (RTD) (BVVL/FL) | *SLC52A2, SLC52A3 (AR)* | 97229 | 607882, 613350 | 79581, 113278 | ID no longer considered a major clinical feature |
| Hypermanganesemia with dystonia type 1 (HMPDC) | *SLC30A10 (AR)* | **309854** | [611146](https://www.omim.org/entry/611146) | [55532](https://hpo.jax.org/app/browse/gene/55532) | ID no longer considered a major clinical feature |
| 3-Methylglutaconic Aciduria Type I | *AUH (AR)* | 67046 | [600529](https://omim.org/entry/600529) | 549 | No longer support for treatment effect on ID or secondary outcomes |
| 2 methyl-3-hydroxybutyryl-CoA dehydrogenase (MHBD) deficiency (HSD10 disease) | *HSD17B10 (X-linked)* | **391417** | 300256 | 3028 | No longer support for treatment effect on ID or secondary outcomes |
| Glucocerebrosidase deficiency (Gaucher Disease Type III) | *GBA (AR)* | **77261** | [606463](https://omim.org/entry/606463) | 2629 | No longer support for treatment effect on ID or secondary outcomes |
| Sanfilippo Syndrome A (MPS IIIa) | *SGSH (AR)* | **79269** | [605270](https://omim.org/entry/605270) | [6448](https://hpo.jax.org/app/browse/gene/6448) | No longer support for treatment effect on ID or secondary outcomes |
| Sanfilippo Syndrome B (MPS IIIb) | *NAGLU (AR)* | **79270** | [609701](https://omim.org/entry/609701) | [4669](https://hpo.jax.org/app/browse/gene/4669) | No longer support for treatment effect on ID or secondary outcomes |
| Sanfilippo Syndrome C (MPS IIIc) | *HGSNAT (AR)* | **79271** | [610453](https://omim.org/entry/610453) | [138050](https://hpo.jax.org/app/browse/gene/138050) | No longer support for treatment effect on ID or secondary outcomes |
| Sanfilippo Syndrome D (MPS IIId) | *GNS (AR)* | **79272** | [607664](https://omim.org/entry/607664) | [2799](https://hpo.jax.org/app/browse/gene/2799) | No longer support for treatment effect on ID or secondary outcomes |
| Fatty aldehyde dehydrogenase deficiency (Sjögren-Larsson syndrome) | *ALDH3A2 (AR)* | **816** | [609523](https://omim.org/entry/609523) | [224](https://hpo.jax.org/app/browse/gene/224) | No longer support for treatment effect on ID or secondary outcomes |
| Sterol-C4-methyl oxidase (SC4MOL) deficiency | *MSMO1 (AR)* | 488168 | [607545](https://omim.org/entry/607545) | [6307](https://hpo.jax.org/app/browse/gene/6307) | No longer support for treatment effect on ID or secondary outcomes |
| Hypoxanthine guanine phosphoribosyltransferase deficiency (Lesch-Nyhan syndrome) | *HPRT1 (X-linked)* | 510 | [308000](https://omim.org/entry/308000) | [3251](https://hpo.jax.org/app/browse/gene/3251) | No longer support for treatment effect on ID or secondary outcomes |
| Hyperammonemia hyperinsulinism syndrome | *GLUD1 (AD)* | 35878 | [138130](https://omim.org/entry/138130) | [2746](https://hpo.jax.org/app/browse/gene/2746) | ID no longer considered caused by metabolic defect |
| Thiamine-responsive encephalopathy | *SLC19A3 (AR)* | 199348 | [606152](https://omim.org/entry/606152) | [80704](https://hpo.jax.org/app/browse/gene/80704) | Duplicate disorder to Biotin-thiamine-responsive basal ganglia disease |

Names of disorders were based on the most recent International Conference on Inherited Metabolic Disorders (ICIMD) classification. IMD = Inherited Metabolic Disorder. OMIM = Online Mendelian Inheritance in Man®. HPO = Human Phenotype Ontology. Mode of inheritance for each gene is denoted as AD = autosomal dominant; AR = autosomal recessive; X-linked (dominant); Mt = mitochondrial.
